# Coherent control of quantum-dot spins with cyclic optical transitions

**DOI:** 10.1038/s41467-026-74590-z

**Published:** 2026-06-24

**Authors:** Zhe Xian Koong, Urs Haeusler, Jan M. Kaspari, Christian Schimpf, Benyam Dejen, Ahmed M. Hassanen, Daniel Graham, Yusuf Karli, Ailton J. Garcia Jr, Melina Peter, Edmund Clarke, Maxime Hugues, Michał Gawełczyk, Armando Rastelli, Doris E. Reiter, Mete Atatüre, Dorian A. Gangloff

**Affiliations:** 1 Cavendish Laboratory, University of Cambridge, Cambridge, United Kingdom; 2 Department of Physics, TU Dortmund University, Dortmund, Germany; 3 Department of Engineering Science, University of Oxford, Oxford, United Kingdom; 4 Institute of Semiconductor and Solid State Physics, Johannes Kepler University, Linz, Austria; 5 EPSRC National Epitaxy Facility, University of Sheffield, Sheffield, United Kingdom; 6 Université Côte d’Azur, Valbonne, France; 7 Institute of Theoretical Physics, Wrocław University of Science and Technology, Wrocław, Poland

**Keywords:** Qubits, Quantum dots, Quantum optics, Magneto-optics

## Abstract

Solid-state spins are promising as interfaces from stationary qubits to single photons for quantum communication technologies. Semiconductor quantum dots have excellent optical coherence, exhibit near-unity collection efficiencies when coupled to photonic structures, and possess long-lived spins for quantum memory. However, the incompatibility of performing optical spin control and single-shot readout simultaneously has been a challenge faced by almost all solid-state emitters. To overcome this, we leverage light-hole mixing to realize a highly asymmetric lambda system in a negatively charged heavy-hole exciton in Faraday configuration. By compensating GHz-scale differential Stark shifts, induced by unequal coupling to Raman control fields, and by performing nuclear-spin cooling, we achieve quantum control of an electron-spin qubit with a *π*-pulse contrast of 97.4% while preserving spin-selective optical transitions with a cyclicity of 471 (50). We demonstrate this scheme for both GaAs and InGaAs quantum dots, and show that it is compatible with the operation of a nuclear quantum memory. Our approach thus enables repeated emission of indistinguishable photons together with qubit control, as required for single-shot readout, photonic cluster-state generation, and quantum repeater technologies.

## Abstract (web summary)

Simultaneous spin control and single shot readout are key to generating photonic resource states and have remained challenging in semiconductor quantum dots. Here, the authors demonstrate high fidelity optical control of an electron spin qubit in GaAs and InGaAs quantum dots while preserving high optical cyclicity for spin-selective transitions.

## Introduction

Quantum network nodes based on efficient, long-lived spin-photon interfaces are essential for distributed quantum information processing^1–3^. Each node consists of an interface between single photons and a spin qubit, and realizing its full functionality requires initializing and reading out the spin state, encoding information via quantum control over the spin, entangling the spin with a photon, and storing this quantum information in a local memory before sending the photon across the optical link^4,5^. These requirements typically reduce to the simultaneous ability to coherently control a long-lived spin ground state, and to trigger spin-selective emission of photons via cyclic transitions. While direct magnetic-dipole control of the ground state spin is possible in several systems with cyclic transitions^6–8^, in many quantum emitter systems, this remains an outstanding problem to integrate with efficient photonic structures^9^, and all-optical means are thus the only viable route for quantum control. However, all-optical control requires linking the spin states via multiple allowed optical transitions, which can conflict directly with the need for cyclic optical transitions.

Amongst quantum emitters, semiconductor quantum dots (QDs) stand out for their exceptional optical coherence and efficiency, thanks to the continued improvement in material quality^10,11^ and photonic technologies^12,13^. They also feature coherent electronic and nuclear spins^14–17^, such that 100-microsecond^18^ and 100-millisecond^19^ coherence times are attainable for GaAs QDs, respectively. While some low-field control of the spin is possible with limited fidelity^20^, addressing and controlling the electronic spin as a high-fidelity qubit typically requires a few-Tesla magnetic field to lift the degeneracy of the electronic spin states beyond the scale of hyperfine and exciton linewidth energies. Under a growth-axis field (Faraday configuration), the presence of spin-specific circularly-polarized cyclic transitions allows for efficient spin-state readout^21,22^ in the single-shot regime^23,24^. However, spin control of a QD in this configuration has not been realized. Direct microwave drive would need to operate in the few to tens of GHz regime and would need to reach Rabi frequencies of tens of MHz at minimum – for nuclear-spin stabilization of the qubit splitting^25^ – corresponding to tens of mT of magnetic flux density at the QD position; this is a formidable challenge for optically active QD systems. The historical and viable approach for spin control in a QD has thus been to apply an in-plane field (Voigt configuration), resulting in a symmetric linearly polarized *Λ* system which allows all-optical coherent control over the spin states ^14,25–29^, reaching high contrast (99.3%^18^) and full-phase control of an electronic spin with GHz-scale Rabi frequency^29^. Despite efforts to selectively enhance a readout transition in this configuration, via the coupling to a cavity^29,30^ or a waveguide^13,31^, only modest cyclicity  ~ 36 has been achieved. The lack of intrinsically cyclic transitions in the Voigt configuration has hampered the simultaneous realization of both spin-selective readout and all-optical spin-control, and has been a major hurdle for QD systems vying for the realization of photonic quantum technologies.

In this work, we demonstrate quantum control of a QD electron-spin qubit in Faraday geometry – for both a GaAs QD and an InGaAs QD. A small light-hole admixture of the heavy-hole trion weakly breaks the full cyclicity of the spin-selective transitions, while maintaining it at a high value of 471 (50) (291 (28)) for the GaAs (InGaAs) QD, and creates a highly asymmetric *Λ* system. Using a narrowband stimulated Raman scheme to drive electron-spin resonance^14^, we account for the GHz-level differential Stark shifts arising from asymmetric Rabi frequencies in the *Λ* system, something otherwise impossible with broadband ps-pulse lasers^26^. With nuclear-spin stabilization of the qubit splitting, we narrow the electron spin resonance to 7(1) MHz (31(3) MHz), corresponding to an inhomogeneous dephasing time ${T}_{2}^{*}=74(11)\,{{\rm{ns}}}$ (${T}_{2}^{*}=17(2)\,{{\rm{ns}}}$), in a GaAs QD (an InGaAs QD) and achieve phase-tuneable control of the electron-spin qubit – sufficient for functional spin-photon entanglement^31^. With the GaAs QD, we achieve up to 273(6) MHz Rabi frequency and a *π*-pulse contrast of 97.4(1)%. We also perform nuclear-spin spectroscopy and demonstrate the compatibility of this control configuration with a nuclear quantum memory ^17^. Our results demonstrate the technical feasibility of combining high-fidelity coherent control of QD spins and single-shot readout, with direct application to the deterministic generation of large entangled photonic states^32,33^.

**Fig. 1:**
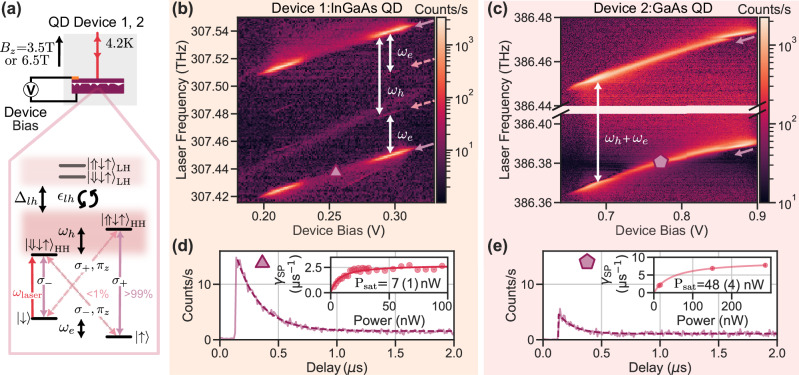
Optical cyclicity of InGaAs and GaAs QDs in Faraday geometry. **a** Top panel: schematic of experimental setup (c.f. Supplementary Note 1) consisting of a charge-tuneable device with bias voltage *V*, containing QDs, under a magnetic field *B*_*z*_ up to 6.5 T along the QD device growth axis *z*. Bottom panel: energy levels of a negatively-charged exciton *X*^1−^ in Faraday configuration for a negative electron *g*-factor^41^. Solid purple arrows show the dominant circularly polarized dipole transitions connecting the electron-spin ground states, $\left|\downarrow \right\rangle$ and $\left|\uparrow \right\rangle$, to the HH trion states, ${\left|\Downarrow \downarrow \uparrow \right\rangle }_{{{\rm{HH}}}}$ and ${\left|\Uparrow \downarrow \uparrow \right\rangle }_{{{\rm{HH}}}}$, respectively. A small admixture (*ϵ*_*lh*_) of the LH *X*^1−^ states (${\left|\Downarrow \downarrow \uparrow \right\rangle }_{{{\rm{LH}}}}$ and ${\left|\Uparrow \downarrow \uparrow \right\rangle }_{{{\rm{LH}}}}$), split to higher energy by *Δ*_*lh*_, with the HH states enables the spin-flipping transitions (pink dashed arrows) to the two electron ground states. **b**, **c** Resonant spectroscopy of the *X*^1−^ of an InGaAs (GaAs) QD at *B*_*z*_ = 3.5 T with a linearly polarized narrow-linewidth laser at a power above saturation,  ~ 2P_sat_ (~ 8 P_sat_), as a function of the device bias *V* and laser frequency. The spin-conserving transitions are highlighted by solid purple arrows, while the spin-flipping transitions are highlighted by pink dashed arrows. For the InGaAs QD (Device 1), we extract an electron splitting *ω*_*e*_ = 30(1) GHz and a hole splitting *ω*_*h*_ = 59(1) GHz from Lorentzian fits to the resonances at *V* = 0.3 V. The combined electron and hole splitting is *ω*_*e*_ + *ω*_*h*_ = 81.6(2) GHz for the GaAs QD (Device 2). **d**, **e** Photon count histogram under time-resolved pulsed resonant excitation at *V* = 0.26 V (*V* = 0.78 V), showing optical spin pumping on the $\left|\downarrow \right\rangle \leftrightarrow \left|\Downarrow \downarrow \uparrow \right\rangle$ transition, indicated by the red arrow in (**a**), at a power above saturation,  ~ 16P_sat_ (~ 6 P_sat_). A decay time of 203(2) ns (111(1) ns) is obtained from an exponential fit (dashed curve). Insets show the spin pumping rate *γ*_SP_ (inverse of decay time) as a function of resonant laser power, where the solid curve is a fit of the data to a saturation model with parameter P_sat_, resulting in P_sat_ = 7(1) nW (P_sat_ = 48(4) nW).

**Fig. 2:**
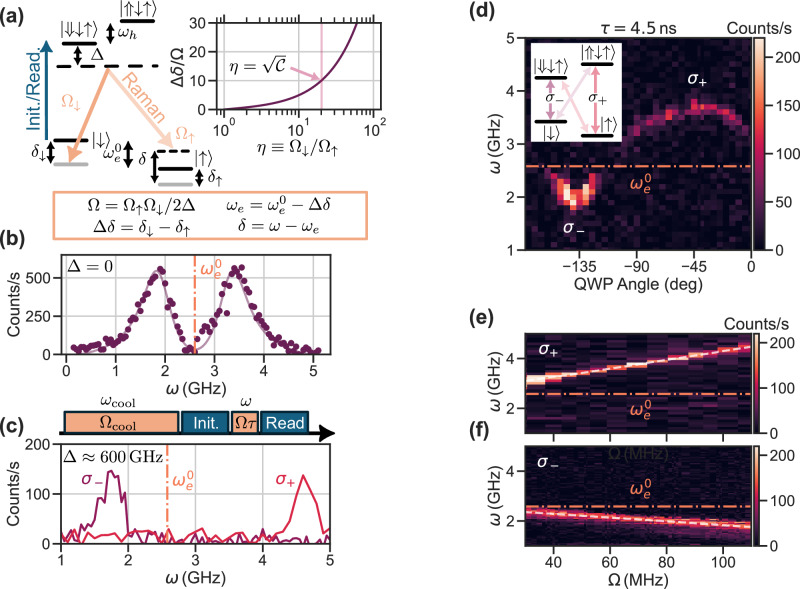
Dependence of electron spin resonance on Raman laser at *B*_*z*_ = 6.5 T. All measurements are taken on a GaAs QD in Device 2. **a** Energy levels relevant for Raman spin control. Each Raman field exerts an a.c. Stark shift (gray lines) *δ*_*↓*_ and *δ*_*↑*_ on its respective ground states. The right panel shows *Δδ*/*Ω* as a function of *η* = *Ω*_*↓*_/*Ω*_*↑*_, as related to cyclicity by $\eta=\sqrt{{{\mathcal{C}}}}$. **b** Continuous two-color resonant (*Δ* = 0) measurement with a fixed-frequency laser (pump) set to 700 nW (≈ 14 P_sat_) and a variable frequency laser (probe) set to 70 nW (≈ 1.4 P_sat_). The resulting fluorescence signal is shown as a function of relative pump-probe (two-photon) detuning *ω*. A fit (solid curve) to the data with a three-level model (see Supplementary Note 7) reveals an electron-spin splitting of ${\omega }_{e}^{0}=2.60\,(1)$GHz from the dark-state (dip) resonance condition $\omega={\omega }_{e}^{0}$. **c** Top: pulse sequence for obtaining an ESR spectrum consisting of nuclear-spin cooling (40 *μs* of Rabi cooling) at Rabi frequency *Ω*_cool_ = *Ω* and drive frequency *ω*_cool_ = *ω* followed by 1.5 μ*s* of spin initialization, a short probe with Rabi frequency *Ω*, drive frequency *ω* and duration *τ*, and a 1.5 μ*s* spin readout. Bottom: readout fluorescence as a function of drive frequency. Measurements are performed at *Ω* ≈ 110 MHz and *τ* = 4.5 ns using the Raman laser polarization settings aligned to *σ*_−_ (purple data) and *σ*_+_ (red data). **d** ESR spectrum as a function of Raman laser polarization set by the quarter-wave-plate (QWP) angle. QWP angle of 0^∘^ is calibrated to correspond to linear horizontal polarization, and the right-hand (*σ*_+_) and left-hand (*σ*_−_) circular polarizations are at  − 45^∘^ and  − 135^∘^, respectively. **e**, **f** ESR spectrum as a function of Rabi frequency for right-hand (left-hand) circular polarization. Dashed lines are linear fits to the frequency corresponding to a fluorescence maximum, with fitted slopes *Δδ*/*Ω* = − 17.2 (6) and *Δδ*/*Ω* = 7.4 (5). Both fits converge to ${\omega }_{e}^{0}=2.58\,(3)$GHz at the limit of vanishing Ω.

**Fig. 3:**
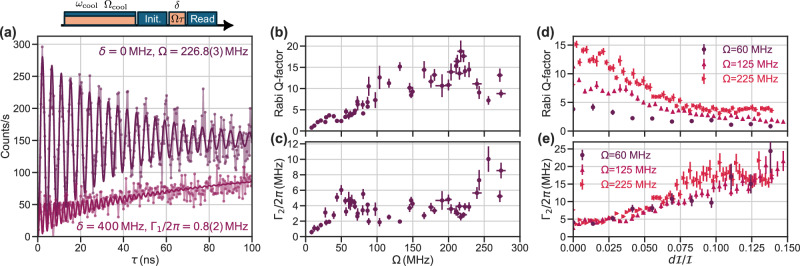
Coherent control of the electron spin. All measurements are taken on a GaAs QD in Device 2. **a** Measurement of Rabi oscillations with pulse sequence (top) consisting of nuclear-spin cooling (Raman cooling) at Rabi frequency *Ω*_cool_ and drive frequency *ω*_cool_ followed by 1.5 μ*s* of spin initialization, a probe pulse with Rabi frequency *Ω*, detuning *δ* and duration *τ*, and a 1.5 μ*s* of spin readout. Measurement data is readout counts as a function of *τ*, and shows Rabi oscillations at *δ* = 0 and laser-induced spin relaxation at *δ* = 400 MHz. The solid curves are a master equation model (Supplementary Note 8), with fixed ${T}_{2}^{*}=34$ ns, used to fit the relaxation rate *Γ*_1_/2*π* = 0.8(2) MHz at *δ* = 400 MHz and the Rabi frequency 226.8(3) MHz and dephasing rate *Γ*_2_/2*π* = 3.7(1) MHz at *δ* = 0 MHz. The Rabi quality factor is *Q* = 15.1(6). **b** Rabi quality factor *Q* and (**c**) spin dephasing rate *Γ*_2_/2*π* as a function of *Ω* for *Ω*_cool_ = 15 MHz. The optical power of the Raman laser entering the cryostat for *Ω* = 250 MHz is approximately 290 μW. **d** Rabi quality factor *Q* and (**e**) spin dephasing rate *Γ*_2_/2*π* as a function of artificially-introduced Raman laser intensity modulation $d{{\mathcal{I}}}/{{\mathcal{I}}}$ at three probe Rabi frequencies *Ω*. Error bars indicate one standard error on fitted parameters.

**Fig. 4:**
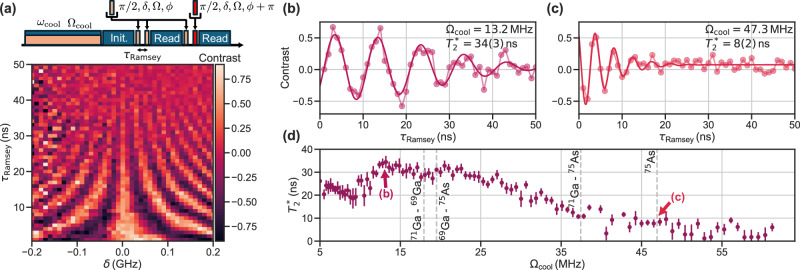
Phase-resolved control of the electron-spin qubit. All measurements are taken on a GaAs QD in Device 2. **a** Ramsey measurement with pulse sequence (top) consisting of nuclear-spin cooling (40 *μs* of Raman cooling) at Rabi frequency *Ω*_cool_ and drive frequency *ω*_cool_ followed by two Ramsey sequences. The first Ramsey sequence consists of 1.5 μ*s* of spin initialization, a first *π*/2 rotation with phase *ϕ* (i.e., our phase reference), a Ramsey delay time *τ*_Ramsey_, a second *π*/2 rotation with phase *ϕ*, and a 1.5 μ*s* of spin readout with average count *n*_*ϕ*_. The second Ramsey sequence consists of spin initialization, a first *π*/2 rotation with phase *ϕ*, a Ramsey delay time *τ*_Ramsey_, a second *π*/2 rotation with phase *ϕ* + *π*, and a spin readout with average count *n*_*ϕ*+*π*_. Our signal is the contrast (*n*_*ϕ*_ − *n*_*ϕ*+*π*_)/(*n*_*ϕ*_ + *n*_*ϕ*+*π*_). All rotations are performed with *Ω* = 125 MHz and a detuning *δ*. Measurement data shows contrast as a function of *δ* and *τ*_Ramsey_ for *Ω*_cool_ = 15 MHz. **b**, **c** Contrast as a function of *τ*_Ramsey_ for *Ω*_cool_ = 13.2 MHz (*Ω*_cool_ = 47.3 MHz). The solid curve is a fitting function $A\sin (2\pi \delta {\tau }_{{{\rm{Ramsey}}}}+\theta )\exp \left(-{\left({\tau }_{{{\rm{Ramsey}}}}/{T}_{2}^{*}\right)}^{2}\right)$ with free parameters: *A* as a contrast amplitude, *δ* as the detuning from ESR, *θ* as a phase, and ${T}_{2}^{*}$ as the characteristic decay time. The relevant fit results are ${T}_{2}^{*}=34(3)\,{{\rm{ns}}}$ $({T}_{2}^{*}=8(2)\,{{\rm{ns}}})$ and *δ* = 100(1) MHz (*δ* = 229(4) MHz). **d** Inhomogeneous dephasing time ${T}_{2}^{*}$, fitted from Ramsey experiments as performed in (**b**, **c**), as a function of *Ω*_cool_. Dashed lines highlight expected single- and double-species nuclear resonances within this range of frequencies. Error bars indicate one standard error on fitted parameters.

**Fig. 5:**
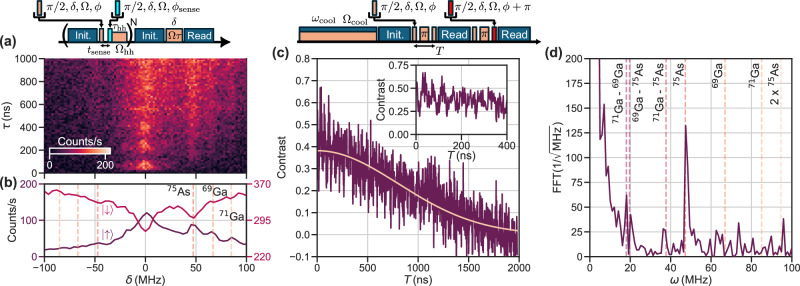
Nuclear-magnon and Hahn-Echo spectroscopy. All measurements are taken on a GaAs QD in Device 2. **a** Nuclear-magnon spectroscopy measurement with pulse sequence (top panel) consisting of nuclear-spin cooling (55 μ*s* of modified quantum algorithmic cooling; see “Methods”) at Rabi frequency *Ω*_hh_ = 47 MHz followed by a probe Rabi sequence consisting of 1.5 μ*s* of spin initialization, a Rabi drive at Rabi frequency *Ω* = 6 MHz, detuning *δ*, and of duration *τ*, and 1.5 μ*s* of spin readout. The color map shows the resulting average readout counts as a function of *δ* and *τ*. **b** Spectrum, acquired as in (**a**), averaged over all Rabi drive durations when the electron spin is initialized to $\left|\uparrow \right\rangle$ (purple), as in (**a**), or to $\left|\downarrow \right\rangle$ (pink), with an additional *π* pulse before the Rabi drive; the readout remains on the $\left|\downarrow \right\rangle$ state. **c** Hahn Echo spectroscopy measurement with pulse sequence (top) consisting of the same pulse sequence as in Fig. 4a with the addition of refocusing *π* pulses halfway between the Ramsey pulses. The resulting contrast as a function of total delay time *T* is shown below. The solid curve is a fit to the function $A\exp \left(-{(T/{T}_{2}^{{{\rm{HE}}}})}^{2}\right)$, where ${T}_{2}^{\,{{\rm{HE}}}}=1.14(2)$ μs and *A* is the amplitude of the envelope. Inset: zoom in of the same data. **d** Fourier transform of the data in (**c**). Dashed lines are expected nuclear Larmor frequencies. Data in (**c**) and (**d**) is taken at a cooling frequency *ω*_cool_ = 2.3 GHz.

**Fig. 6:**
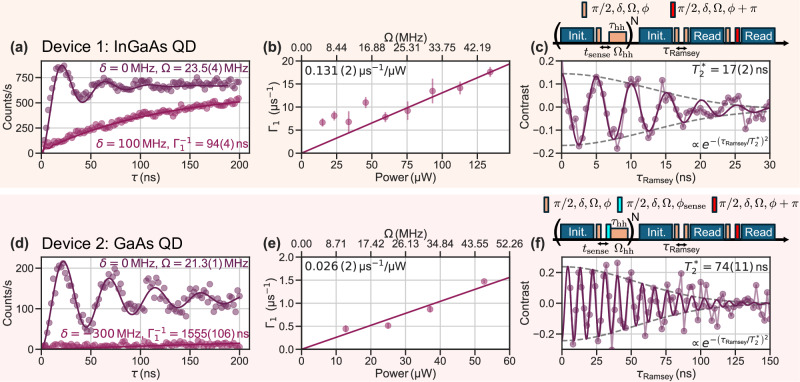
Faraday quantum control of an electron-spin qubit in an InGaAs QD and a GaAs QD. **a**–**f** are data for an InGaAs QD in Device 1 (a GaAs QD in Device 2). Details on quantum-algorithmic cooling used with Device 1 can be found in Supplementary Note 5. **a**, **d** Using the pulse sequence in Fig. 3a with quantum-algorithmic cooling (Raman cooling), Rabi oscillations at *δ* = 0 and laser-induced relaxation at *δ* = 100 MHz (*δ* = − 300 MHz). Solid curves are from our master equation model (Supplementary Note 8) from which we fit *Ω* = 23.5(4) MHz (*Ω* = 21.3(1) MHz) and ${\Gamma }_{1}^{-1}=94(4)\,{{\rm{ns}}}$ (${\Gamma }_{1}^{-1}=1555(106)$ ns). **b**, **e** *Γ*_1_ as a function of Raman laser power. The solid line is a linear fit (no offset) with slope 0.131(2)μs^−1^/μW (0.026(2)μs^−1^/μW). **c**, **f** Our pulse sequence consists of quantum-algorithmic cooling, which for Device 2 (**f**) is adapted to the transverse collinear interaction by adding a *π*/2 pulse with a phase *ϕ*_sense_ (see “Methods”). This is followed by a Ramsey measurement as done in Fig. 4, but with a serrodyne frequency *f*_serr_ = 200 MHz (100 MHz) applied to the second Ramsey pulse. The data show Ramsey contrast as a function of Ramsey delay *τ*_Ramsey_. The solid curve is a fitting function $A\sin (2\pi f\tau )\exp (-{(\tau /{T}_{2}^{*})}^{2})$ with free parameters: *A* as the contrast amplitude, *f* ≈ *f*_serr_ as the contrast oscillation frequency, and ${T}_{2}^{*}$ as the characteristic decay time. The fit results are ${T}_{2}^{*}=17(2)\,{{\rm{ns}}}$ (${T}_{2}^{*}=74(11)$ ns), $f=\left(197(2)\right.\,{{\rm{MHz}}}$ (*f* = 112(1) MHz). Error bars indicate one standard error on fitted parameters.

Measurements presented in Fig. 1 are taken on both an InGaAs QD and a GaAs QD. Measurements presented in Figs. 2, 3, 4, and 5 are taken on a GaAs QD, while a subset of similar measurements performed on an InGaAs QD are presented in Supplementary Note 5. Figure 6 provides a summary on the electron-spin measurements taken on both an InGaAs QD and a GaAs QD.

## Results

### Devices and spectroscopy

We use two different QD devices in this work (Fig. 1a). The first consists of self-assembled InGaAs QDs emitting around 975 nm embedded in a Schottky diode (Device 1), while the second consists of droplet-etched GaAs QDs emitting around 775 nm embedded in a *p-i-n* diode (Device 2). Both devices are kept cold in a cryostat at 3.5–4.2 K, and are subject to a magnetic field up to *B*_*z*_ = 8.0 T applied along the growth axis (*z*) of the QDs. Details on the design of both devices can be found in Supplementary Note 1.

For both devices, we work with a single electron-charged QD, consisting of two Zeeman-split ground electron-spin states $\left|\downarrow \right\rangle$ and $\left|\uparrow \right\rangle$, and two excited negatively-charged exciton (trion) *X*^1−^ states $\left|\Downarrow \downarrow \uparrow \right\rangle$ and $\left|\Uparrow \downarrow \uparrow \right\rangle$. The trion hole states $\left|\Uparrow \right\rangle$ and $\left|\Downarrow \right\rangle$ consist predominantly of heavy-hole (HH) states with a small admixture of light-hole (LH) states^34,35^ – as parametrized by the HH-LH admixture coefficient *σ*_*lh*_/Δ_*lh*_ ≡ *ϵ*_*lh*_ ≪ 1, where *σ*_*lh*_ parametrizes the off-diagonal coupling (in the Luttinger-Kohn Hamiltonian) – due to strain, disorder, or confinement effects – between LH and HH states and Δ_*lh*_ is the HH-LH energy splitting. The energy level schematic, depicted in Fig. 1a, highlights the dominant circularly-polarized spin-conserving transitions $\left|\Downarrow \downarrow \uparrow \right\rangle \leftrightarrow \left|\downarrow \right\rangle$ and $\left|\Uparrow \downarrow \uparrow \right\rangle \leftrightarrow \left|\uparrow \right\rangle$ and the weaker spin-flipping transitions, $\left|\Downarrow \downarrow \uparrow \right\rangle \leftrightarrow \left|\uparrow \right\rangle$ and $\left|\Uparrow \downarrow \uparrow \right\rangle \leftrightarrow \left|\downarrow \right\rangle$. The spin-flipping transitions are forbidden for a pure HH *X*^1−^ states and weakly enabled in the presence of HH-LH admixture^36^. Depending on the exact nature of the LH mixing, the spin-flipping transitions can be linear *π*_*z*_ – i.e., along the growth, magnetic-field, and optical axes, and thus mostly dark^37^ – or circularly polarized (see Supplementary Note 2). Despite the large strains present in InGaAs QDs, the spin-conserving HH transitions remain approximately cyclic due to the small QD sizes and thus to a large value of Δ_*lh*_ in the tens of meV^38^. While typically larger than 100: 1, the exact branching ratio between the spin-conserving and spin-flipping transitions can vary significantly across multiple QDs in the device due to the strain inhomogeneity of the Stranski-Krastanov growth process^35,39^. The origin of the non-zero HH-LH admixture in a typically strain-free GaAs QD can be attributed to 3D confinement effects in nanostructures^40^ – a smaller effective Δ_*lh*_ of  ~ 3 meV together with small geometry- or disorder-induced effective shear strain components at the QD material interface^41^. Figures 1b–e present the optical spectroscopy of the *X*^1−^ transition in an InGaAs QD (Device 1) and a GaAs QD (Device 2) at *B*_*z*_ = 3.5 T. A voltage bias *V* is applied to the Schottky (Device 1) or to the p-i-n (Device 2) diode to enable charge control and tuning of the emission energy via the d.c. Stark shift. Using a dark-field microscope^42^, we scan across the *X*^1−^ resonance with a linearly polarized narrow-linewidth (~ 100 kHz) laser. The collected fluorescence from Device 1 (Device 2) is shown in Fig. 1b (Fig. 1(c) as a function of device bias and laser frequency, at laser powers well above saturation. At the edge of the charging plateau (device bias of  ≈ 0.22 V and  ≈ 0.30 V for Device 1 and device bias of  ≈ 0.68 V and  ≈ 0.87 V for Device 2), rapid tunneling of the electron from the QD to the diode back contact reservoir randomizes the spin states^43^, thus preventing spin pumping and allowing us to observe steady-state fluorescence on resonance. In Device 1, we observe four distinct resonances: two strong and two weak transitions with relative intensities of approximately 100: 1. This is consistent with the level diagram of Fig. 1a, where the two strong (weak) transitions correspond to the spin conserving (flipping) transitions, and demonstrates that it is possible to directly excite the normally forbidden spin-flipping transitions. We further extract an electron-spin splitting of 30 GHz and a trion linewidth of 471(36) MHz from a Lorentzian fit to all four peaks at this bias. For Device 2, we observe only two sharp resonances, corresponding to the spin-conserving transitions; this is likely due to the small electron-spin splitting – a few GHz for GaAs QDs at this wavelength and field^41^ – making the direct excitation of weak spin-flipping transitions difficult to resolve in close proximity to the strong spin-conserving transitions. The small spin splitting of the GaAs QD is also responsible for the reduced contrast, relative to the InGaAs QD, between the edges and the center of the charging plateau (see Supplementary Note 1). The splitting between the two spin-conserving transitions reveals a combined electron and hole splitting of *ω*_*h*_ + *ω*_*e*_ = 81.6(2) GHz. For this QD, we measure a trion linewidth of 851(27) MHz from a Lorentzian fit to the data taken at a device bias of 0.75 V and at zero field. The discontinuity in the GaAs QD trion line in top right of Fig. 1c is most likely due to the exponential dependence of the spin relaxation rate on the bias close to the co-tunneling region^44^: in the middle of the plateau the highest steady-state fluorescence appears at a bias which maximizes off-resonant pumping and repumping (possible for a small spin splitting), whereas at the edges where spin relaxation is much faster than repumping it appears at a bias corresponding to resonant scattering (similar though less marked features can be seen on all branches on the approach to the co-tunneling zone).

Focusing now on a device bias in the middle of the charging plateau, we observe a significantly lower intensity from the dominant peaks compared to that at the edge of the plateau (Fig. 1b, c). Despite the weakness of the spin flipping process in this field configuration (allowed by HH-LH mixing^41^), this measurement confirms the presence of significant optical pumping of the electron-spin population against background spin relaxation mechanisms. A measurement of spin relaxation time *T*_1_, exceeding 40 μs for both devices, is presented in Supplementary Note 1. We then probe the time scale of the spin-pumping process by measuring the time it takes for the electron spin to initialize to $\left|\uparrow \right\rangle$ by continuously driving the $\left|\downarrow \right\rangle \leftrightarrow \left|\Downarrow \downarrow \uparrow \right\rangle$ transition for 5 *μs*, shown in Fig. 1d, e for the two devices, respectively. Taken with a readout power of 16 (6) times the saturation power *P*_sat_, to approach the minimum pumping time, our measurement reveals a fitted exponential decay time of ${\gamma }_{\,{{\rm{SP}}}}^{-1}=203(2)\,{{\rm{ns}}}$ (${\gamma }_{\,{{\rm{SP}}}}^{-1}=111(1)\,{{\rm{ns}}}$) for Device 1 (2), yielding the maximum spin-flipping scattering rate *γ*_SP_. The steady-state fluorescence at long delay time is dominated by off-resonant repumping of the spin state by the readout laser at these high powers. For this reason, in subsequent measurements presented in this article for both Devices 1 and 2, we perform spin initialization at lower powers of 5*P*_sat_ and 0.5*P*_sat_ (and for Device 2 at a higher magnetic field) for which repumping is no longer significant and an initialization fidelity upper bound of 98% and 97% can be reached, respectively (as detailed in Supplementary Note 1). Using the previously reported *X*^1−^ lifetime of ${\gamma }_{1}^{-1}=0.696\,(65)\,{{\rm{ns}}}$^45,46^ for an InGaAs QD on this device and an estimated lifetime of ${\gamma }_{1}^{-1}=0.235\,(25)$ ns (c.f. Supplementary Note 6) for the GaAs QD, we obtain an optical cyclicity of ${{\mathcal{C}}}\equiv {\gamma }_{{{\rm{SC}}}}/{\gamma }_{{{\rm{SP}}}}={\gamma }_{1}/{\gamma }_{{{\rm{SP}}}}-1=291\,(28)$ and 471 (50), where *γ*_SC_ is the spin-conserving scattering rate, and a branching ratio for the spin-flipping transition *γ*_SP_/*γ*_1_^43,47^ of 0.34 (3)% and 0.21 (2) %, respectively. The cyclicity ${{\mathcal{C}}}$ determines the mean number of photons scattered by the bright spin state before it flips to the dark spin state during a readout event, and sets the upper limit for the single-shot readout fidelity^48^.

### Stimulated Raman transitions in Faraday configuration

Figure 2 (a) shows the relevant energy levels involved in our spin control experiment. For readout, a resonant laser drives the lowest energy cycling transition $\left|\Downarrow \downarrow \uparrow \right\rangle \leftrightarrow \left|\downarrow \right\rangle$ at 0.5 P_sat_(≈ 25 nW) for both initialization to the spin $\left|\uparrow \right\rangle$ state and readout of the $\left|\downarrow \right\rangle$ state. To coherently control the ground state spin, we use an all-optical Raman drive that is circularly polarized and red-detuned by the single-photon detuning Δ ≈ 600 GHz from the $\left|\Downarrow \downarrow \uparrow \right\rangle$ trion. The two Raman fields are derived from microwave-driven electro-optical modulation of a single narrow-band continuous-wave laser, resulting in a coherent pair of fields ${\vec{E}}_{\downarrow }$ and ${\vec{E}}_{\uparrow }$ equal in both intensity and polarization^27^. The two Raman fields, with resonant Rabi frequencies Ω_*↓*_ and Ω_*↑*_, respectively, induce a rotation of the ground-state spin at a two-photon Rabi frequency Ω = Ω_*↓*_Ω_*↑*_/2Δ when their frequency difference, the two-photon detuning *ω*, matches the ground-state splitting *ω*_*e*_, i.e., achieving electron-spin resonance (ESR) at *δ* ≡ *ω* − *ω*_*e*_ = 0. The small branching ratio of the spin-flipping transition implies a large imbalance in the Rabi frequencies of the two transitions – Ω_*↓*_ ≫ Ω_*↑*_ (Ω_*↓*_ ≪ Ω_*↑*_) for left-circularly (right-circularly) polarized Raman beams – which results in a large differential (a.c.) Stark shift $\Delta \delta \equiv {\delta }_{\downarrow }-{\delta }_{\uparrow }=({\Omega }_{\downarrow }^{2}-{\Omega }_{\uparrow }^{2})/4\Delta$ on the electron-spin states during the Raman drive. We thus tune *ω* to match the ground state spin splitting ${\omega }_{e}={\omega }_{e}^{0}-\Delta \delta$, where ${\omega }_{e}^{0}$ is the bare electron-spin splitting, and probe Raman resonance (*ω* ~ *ω*_*e*_) when driven.

To visualize the effect of this imbalance, we can express the two-photon Rabi frequency Ω and the differential Stark shift Δ*δ* in terms of Ω_*↓*_ and the Rabi frequency imbalance, *η* ≡ Ω_*↓*_/Ω_*↑*_ as

1 $$\Omega=\frac{{\Omega }_{\downarrow }{\Omega }_{\uparrow }}{2\Delta }=\frac{{\Omega }_{\downarrow }^{2}}{2\eta \Delta }$$

and

2 $$\Delta \delta=\frac{{\Omega }_{\downarrow }^{2}-{\Omega }_{\uparrow }^{2}}{4\Delta }=\Omega \frac{{\eta }^{2}-1}{2\eta },$$

respectively. In the situation where the two Raman fields have equal intensity, $| {\vec{E}}_{\downarrow }{| }^{2}=| {\vec{E}}_{\uparrow }{| }^{2}$, and are exactly matched to two transition dipoles ${\vec{d}}_{\downarrow }$ and ${\vec{d}}_{\uparrow }$ (i.e., the expectation value of the electric dipole Hamiltonian $\langle {\vec{d}}_{\cdot }\vec{E}\rangle$ is maximized),

3 $$\begin{array}{l}{\eta }^{2}={\left(\frac{{\Omega }_{\downarrow }}{{\Omega }_{\uparrow }}\right)}^{2}={\left(\frac{\langle {\vec{d}}_{\downarrow }\cdot {\vec{E}}_{\downarrow }\rangle }{\langle {\vec{d}}_{\uparrow }\cdot {\vec{E}}_{\uparrow }\rangle }\right)}^{2}\\ \to {\left(\frac{| {\vec{d}}_{\downarrow }| }{| {\vec{d}}_{\uparrow }| }\right)}^{2}=\frac{{\gamma }_{{{\rm{SC}}}}}{{\gamma }_{{{\rm{SP}}}}}={{\mathcal{C}}},\end{array}$$

(or $={\gamma }_{{{\rm{SP}}}}/{\gamma }_{{{\rm{SC}}}}={{{\mathcal{C}}}}^{-1}$) for left-circular (right-circular) polarization. Using our measured cyclicity of ${{\mathcal{C}}}=471\,(50)$, we can thus expect a shift in the ESR resonance by 11(1) Ω. At a typical Rabi frequency of Ω = 100 MHz, this corresponds to shifts of ∣Δ*δ*∣ ≃ 1 GHz, which exceeds even the inhomogeneous ESR linewidth (~ 300 MHz^14,18,49^).

As we are unable to resolve the electron splitting via spectroscopy for the GaAs QD in Device 2 (Fig. 1c), we perform two-laser resonant spectroscopy at a fixed bias of 0.775 V and a magnetic field of 6.5 T with a fixed pump laser frequency at 386.435 THz while varying the frequency of a second probe laser. The pump and probe laser powers are fixed at approximately 14 P_sat_ and 1.4 P_sat_, respectively. The resulting steady-state fluorescence data is shown in Fig. 2b and shows a dip in the signal, a signature of coherent population trapping (CPT)^50–52^, when the two lasers meet the two-photon resonance condition for the spin-conserving and spin-flipping transitions. Following a fit to the measurement data using the model and parameters described in Supplementary Note 7, the dip minimum reveals a bare electron splitting of ${\omega }_{e}^{0}=2.60(1)$GHz – corresponding to an out-of-plane *g*-factor of 0.0286(1), as consistent with previous measurements^41^.

Finding the ESR frequency in a Raman experiment typically requires stabilizing the Overhauser shift imparted by nuclear spins on the electron-spin splitting^14^. We do this using nuclear-spin cooling techniques – one of Raman^14^, Rabi^49^, or quantum-algorithmic^25^ cooling, depending on the experiment. Our typical pulse sequence, shown in Fig. 2c, consists of a cooling period – during which our coherent Raman drive and resonant readout serve to sense and correct the nuclear polarization – followed by a probe experiment (representing  ~ 10% of the duty cycle) in which the electron spin is initialized, driven coherently by Raman beams for a fixed duration, and read out. Fixing the probe Rabi frequency to Ω ≈ 110 MHz and the pulse duration to *τ* = 1/(2Ω) = 4.5 ns, i.e., *π* pulse, we measure the readout fluorescence as a function of probe frequency *ω* – here the Raman cooling frequency *ω*_cool_ is also set to *ω*. We observe a clear electron-spin resonance (ESR) signal shifted from ${\omega }_{e}^{0}$ by about  ~ 1 GHz (2 GHz) down (up) for left-circularly (right-circularly) polarized Raman light. The sign of the shift matches our expectation as a red-detuned beam lowers the energy of the ground state; here, the stronger spin-conserving left-circularly (right-circularly) polarized transition lowers the *↓* (*↑*) state more than the spin-flipping transition lowers the state *↑* (*↓*), resulting in a decrease (an increase) in the electron-spin splitting that is proportional to the Raman laser intensity. Figure 2d shows a summary of this measurement as a function of the angle of the quarter-wave plate (QWP) used to tune the circularity of the Raman laser, where 0^*o*^ is calibrated to correspond to linear horizontal polarization. We observe clearly that the Raman laser must exhibit a well-defined handedness for ESR to be possible. Moreover, the maximal amplitude and maximally-positive and negative shifts in the ESR resonance occur at  − 45^*o*^ and  − 135^*o*^, respectively, corresponding to *σ*_+_ and *σ*_−_ polarization settings and matching the dipoles of the two spin-conserving transitions (further details in Supplementary Note 3). This implies destructive interference of the two *Λ* systems when driving with linearly polarized light, which is expected for a specific kind of HH-LH mixing as shown in Supplementary Note 2– by contrast, on the InGaAs QD in Device 1 a degree of linear diagonal polarization seems to improve the Raman coupling (see Supplementary Note 5), which implies a different kind of HH-LH mixing (see Supplementary Note 2). We further observe a larger maximal shift from the undriven resonance frequency ${\omega }_{e}^{0}$ (horizontal dashed line) for *σ*_+_ than for *σ*_−_, consistent with the difference in ESR shifts in Fig. 2c, and a clear deviation from a simple sinusoidal dependence on the QWP angle. While we expect a small difference in coupling between *σ*_+_ and *σ*_−_ polarizations, due to the trion splitting making the Raman detuning for *σ*_+_ larger by *ω*_*h*_ ≈ 150 GHz, this asymmetric response to *σ*_+_ and *σ*_−_ suggests an additional mechanism is present.

Further evidence for this is provided by Fig. 2(e,f), which shows a summary of ESR measurements now taken as a function of probe Rabi frequency Ω, for right-circular (e) and left-circular polarization (f). We observe a clear linear dependence on Ω of the ESR peak frequency ${\omega }_{e}={\omega }_{e}^{0}-\Delta \delta$, as expected from Eqn. (2). Fitting *ω*_*e*_ as a function of Ω with two independent linear functions, we obtain Δ*δ*/Ω = − 17.2(6) and Δ*δ*/Ω = 7.4(5), corresponding to Rabi frequency imbalances of *η*^−1^ = 34(1) and *η* = 15(1), respectively, and an undriven resonance frequency ${\omega }_{e}^{0}=2.58(3)$ GHz that agrees closely between the two linear fits (and is within one standard error of our CPT result in Fig. 2b. These values of *η* deviate measurably from the idealized value derived from our measured optical cyclicity $\eta=\sqrt{{{\mathcal{C}}}}=22(1)$. Equations (1) and (2) tell us that the differential Stark shift is dominated by the spin-conserving transition Rabi frequency Ω_*↓*_ (Ω_*↑*_) for *σ*_−_ (*σ*_+_), whereas the two-photon Rabi frequency Ω is equally affected by the spin-conserving and spin-flipping contributions. The unequal response of the ESR frequency as a function of Rabi frequency between *σ*_+_ and *σ*_−_ polarizations thus reveals that the Raman beam coupling to the spin-flipping transition must differ significantly between the two polarization settings.

We propose the following explanation for this asymmetric response to *σ*_+_ and *σ*_−_, as further detailed in Supplementary Note 2. In general, the LH admixtures to the $\left|\Uparrow \right\rangle$ and $\left|\Downarrow \right\rangle$ are not equal in a magnetic field, an effect which is more pronounced for GaAs QDs due to a lower value of Δ_*lh*_. The kinetics of the two lambda systems are thus asymmetric, rendering *η* and ${{\mathcal{C}}}$ dependent on the helicity and magnetic-field strength. A simple evaluation of the hole-spin-dependent value of Δ_*lh*_ in a 6.5 T field, using known GaAs values for the LH and HH g-factors, yields a predicted ratio between *η*^−1^ for *σ*_+_ and *η* for *σ*_−_ of  ~ 2, which agrees well with our measured ratio of 2.3(2). This asymmetry reveals a potentially useful feature for GaAs QDs at high magnetic fields: that there is a transition with enhanced cyclicity – here *σ*_+_ with ${{\mathcal{C}}}={\eta }^{-2} \sim 1100$ – best suited for readout, and another with reduced cyclicity – here *σ*_−_ with ${{\mathcal{C}}}={\eta }^{2} \sim 225$ – best suited for spin control and spin initialization.

The results in Fig. 2 highlight the essence of optical spin control in the Faraday configuration: an inevitable shift in the ESR frequency that depends on Raman laser polarization and the instantaneous Rabi frequency. If a *π*_*z*_ component is present in the spin-flipping transition (as we suppose is the case for the InGaAs QD but not the GaAs QD), then this shift could be mitigated, though not eliminated, with an asymmetric Raman drive. In all instances, it must be calibrated, as we have done, before experiments with programmable pulse amplitude and phase can be performed.

### Rabi oscillations and quality factor

With the stabilization of the ESR and the calibration of the differential Stark shift achieved, we proceed to show time-resolved coherent driving of the electron spin on resonance. For that, we measure the readout counts as a function of probe pulse duration following stabilization of the nuclei via Raman cooling, as depicted in Fig. 3a. The resulting signal shows damped Rabi oscillations when the ESR drive is resonant (*δ* = 0). When the detuning is increased to *δ* = 400 MHz, we observe a monotonic increase in bright-state population once a few detuned Rabi oscillations have decohered (*τ* ≳ 30 ns); an exponential fit yields a relaxation rate of *Γ*_1_/2*π* = 0.8(2) MHz and provides a direct measurement of background optically induced spin relaxation^27^. The purple solid curve is a fit to a master equation model (see Supplementary Note 8) accounting for the measured inhomogeneous broadening of 14 MHz (${T}_{2}^{*}=34$ ns), the measured spin relaxation rate *Γ*_1_, and spin dephasing with a free parameter *Γ*_2_. From this fit, we extract a Rabi frequency of Ω = 226.8(3) MHz (or a *π*-time of *t*_*π*_ = 1/2Ω = 2.195(2) ns) and spin dephasing rate of *Γ*_2_/2*π* = 3.7(1) MHz. From the fitted density matrix, we also extract a *π*-pulse contrast of 96.8(1)%, which is an upper bound on the *π*-pulse fidelity^27^, and a corresponding Rabi oscillation quality factor $Q=-1/ln(2{f}_{\pi }-1)=15.1(6)$. Figure 3b, c shows the quality factor *Q* and spin dephasing rate *Γ*_2_, extracted from our master equation model as in Fig. 3a, as a function of probe Rabi frequency Ω. Our optimal factor of *Q* = 18.8(3), corresponding to a *π*-pulse contrast of 97.4(1)%, is obtained for Ω = 217(5) MHz. The overall increase in *Q* towards this value is expected from previous work^27,49^, where Rabi frequencies well beyond ${(2\pi {T}_{2}^{*})}^{-1}$ and nuclear Zeeman frequencies (50 − 80 MHz) enable decoupling from nuclear effects. This behavior is also reflected in our fitted *Γ*_2_ values, which start out low due to inhomogeneity-dominated dephasing at low Ω, peak at 4 − 6 MHz around Ω = 50 MHz (i.e., the Arsenic nuclear Larmor frequency) and taper off to 2 − 4 MHz at higher Ω values. The subsequent decrease in *Q* and increase in *Γ*_2_ for Ω ≳ 230 MHz, however, is likely due to a mechanism made worse by Faraday-geometry spin control: laser intensity noise. Any fluctuation in Raman laser intensity not only changes the instantaneous Rabi frequency but also the electron-spin splitting directly via the differential Stark shift. As Ω scales linearly with laser intensity, ${{\mathcal{I}}}$, we can determine the fluctuation in the ESR shift as $d(\Delta \delta )/\Delta \delta=d{{\mathcal{I}}}/{{\mathcal{I}}}$. In the experiment, we measured a typical laser power fluctuation  ~ 1 % (integrated over the full bandwidth of our photo-detector) that worsened at our maximum laser intensities. With a differential Stark shift of Δ*δ* = 7.4(5) Ω (see Fig. 2f), Ω = 250 MHz would result in a broadening of the ESR resonance *d*(Δ*δ*) ≲ 18.5 MHz. While our fitted dephasing rate of *Γ*_2_/2*π* = 8 − 10 MHz for Ω ≈ 250 MHz is significantly lower than this bound, this could be explained by a partial decoupling from the noise spectrum below Ω in a resonant Rabi experiment^27^. To verify this further, we artificially introduce broadband intensity noise (up to 35 MHz) on the Raman laser and measure the resulting Rabi oscillations. The summary is shown in Fig. 3d, e, where a steady decrease of *Q* and a steady increase of *Γ*_2_, respectively, as a function of $d{{\mathcal{I}}}/{{\mathcal{I}}}$ can be observed for $d{{\mathcal{I}}}/{{\mathcal{I}}}\gtrsim 0.025$. These behaviors are qualitatively matched by our master equation model (see Supplementary Note 4), which treats the added intensity noise as a source of inhomogeneity.

### Quantum control and coherence

Next, we demonstrate complete control over the electron-spin qubit by performing phase-modulated Ramsey interferometry. Our sequence, shown in Fig. 4a, consists of two sequences, one with and one without an extra *π* phase on the second *π*/2 pulse, which follows a delay time *τ*_Ramsey_ from a first *π*/2 pulse. This two-sequence configuration thus reads out both spin states at every delay time, providing a balanced readout. The readout counts are given by *n*_*ϕ*_ and *n*_*ϕ*+*π*_, respectively, where *ϕ* is the pulse phase, and our signal is then the contrast (*n*_*ϕ*_ − *n*_*ϕ*+*π*_)/(*n*_*ϕ*_ + *n*_*ϕ*+*π*_). Figure 4a shows the Ramsey contrast as a function of drive detuning *δ* and Ramsey delay *τ*_Ramsey_, displaying the expected chevron pattern in the presence of sufficient coherence to observe clearly resolved fringes.

To measure the inhomogeneous dephasing time ${T}_{2}^{*}$ we take the Ramsey data at a finite detuning *δ* from ESR, corresponding to a Ramsey-delay-dependent phase *ϕ* + 2*πδτ*_Ramsey_ on the second *π*/2 pulse (*ϕ* as our phase reference for the first pulse), and thus contrast oscillations at a frequency *δ* to enable better fitting to the decay envelope. This is shown in Fig. 4b, where we extract ${T}_{2}^{*}=34(3)\,{{\rm{ns}}}$ from a Gaussian envelope fit with *δ* = 100(1) MHz. As known from previous work^14^, the dephasing time ${T}_{2}^{*}$ depends on the cooling Rabi frequency Ω_cool_ – as shown by a striking decrease to ${T}_{2}^{*}=8(2)\,{{\rm{ns}}}$ for Ω_cool_ = 47.3 MHz (Fig. 4c). A full summary of the dependence of ${T}_{2}^{*}$ on Ω_cool_ is shown in Fig. 4d, where the optimal cooling performance is found for Ω_cool_ ~ 15 MHz. This is consistent with previous results in the Raman cooling configuration, where the optimal cooling Rabi frequency was a fraction of the nuclear Zeeman frequency^14^.

### Origins of nuclear cooling

To probe the nature of the electro-nuclear interaction, we run nuclear-magnon spectroscopy^14^, which consists of a Rabi probe sequence where the drive detuning and duration are varied, while the drive Rabi frequency is fixed to Ω = 6 MHz. The results are shown in Fig. 5a, b, where at *δ* ≈ 0 we observe our typical ESR signal and Rabi oscillations, with a full-width at half-maximum of 7(1) MHz consistent with ${T}_{2}^{*}=74(11)\,{{\rm{ns}}}$ – obtained with modified quantum algorithmic cooling (see Methods). At a finite detuning *δ* ≈ 47 MHz, which matches the Arsenic (As) Larmor frequency, we see another resonance appear, corresponding to a magnon mode of that species. Less pronounced resonances at *δ* ≈ 67 MHz and *δ* ≈ 85 MHz likely correspond to the two Gallium isotopes, ^69^Ga and ^71^Ga. This confirms our ability to excite nuclear magnon modes in this Faraday configuration, as a first step towards a quantum memory^17^.

The presence of nuclear resonances is further confirmed by performing Hahn Echo spectroscopy^53,54^, where our probe sequence consists of the addition of a refocusing *π* pulse half-way between the Ramsey pulses of Fig. 4a. Figure 5b shows the contrast as a function of total delay time *T*. The overall decaying contrast as a function of *T* is fitted to a Gaussian function to extract the electron-spin qubit decoherence time ${T}_{2}^{\,{{\rm{HE}}}}=1.14(2)$ μs – shorter than previous results on GaAs^16,18^, which is consistent with a stronger interaction with the transverse nuclear field owing to a QD whose physical size is smaller (blue-shifted^41^) and whose electron-spin splitting ${\omega }_{e}^{0}$ is smaller. Zooming in on short delays, shown in the inset of Fig. 5b, we see oscillations of the contrast, which a Fourier transform of the signal, shown in Fig. 5c, confirms to be predominantly at the As Larmor frequency. These Hahn Echo oscillations and the magnon resonances are features of an interaction between the electron spin and a precessing transverse nuclear field^16,55^, which allows the electro-nuclear energy exchange required for nuclear-spin cooling^25^ and quantum gates between the electron-spin qubit and a nuclear magnon^17^.

For an InGaAs QD, strain produces a large misalignment of nuclear and electronic quantization axes that, independently of the magnetic field direction due to strain inhomogeneity, naturally leads to a *non-collinear* hyperfine interaction^56^ as used in cooling^14^. For a strain-free GaAs QD, a non-collinear interaction can arise from electronic g-factor anisotropy when the magnetic field is not aligned with the crystal axes of GaAs^16^. When subject to a growth-axis magnetic field, however, as is the case here, the electronic and nuclear quantization axes should be fully aligned, and there should not be a significant non-collinear interaction. Instead, the small electron-spin splitting ${\omega }_{e}^{0}=2.6$ GHz makes the transverse *collinear* interaction, whose amplitude scales as ${\omega }_{e}^{-1}$, sufficiently strong to be observed here. A hallmark of this interaction is that it enables only spin-conserving processes and thus only a single sideband in the ESR spectrum – unlike the non-collinear interaction, which allows bidirectional sideband processes^14,56^. This dominant sideband is precisely what we observe in Fig. 5b, where its blue-detuning (*δ* > 0) is seen to be independent of the probe electron-spin state as consistent with a single spin-conserving process. We note the presence of a weaker red-detuned (*δ* < 0) sideband near the As frequency (Fig. 5a, b) – suggesting a small non-collinear interaction is present, possibly due to residual strain or to a slight misalignment of the magnetic field with respect to the device – which allows some degree of bidirectional feedback under the Raman cooling configuration (Fig. 4), despite the asymmetry^57^.

### Control of QD spins

Lastly, we offer a succinct performance overview of InGaAs (Device 1) and GaAs (Device 2) QDs in Fig. 6, where Rabi oscillations and laser-induced relaxation rate (at the same Rabi frequency), and Ramsey interferometry are shown side-by-side. Laser-induced relaxation – which we know from previous work to be purely power-dependent and not related to scattering from the trion^27,58^ – is measured as a function of Raman laser power and fitted to 0.131(2) μs^−1^/μW for Device 1 (which is  ~ 25% of what was previously measured on the same wafer^27^) and to 0.026(2) μs^−1^/μW for Device 2, which is to the best of our knowledge the lowest value reported for any InGaAs or GaAs spin-control experiment. While the lower rate of laser-induced relaxation for Device 2 is a contributor to higher quality Rabi oscillations, the dominant dephasing mechanism for both devices is a *T*_2_ process that likely arises in part from laser intensity noise coupled in via the differential Stark effect.

For Device 1, our best Rabi oscillations reach Ω = 66(7) MHz and *Q* = 0.90(8), and our best measured coherence is ${T}_{2}^{*}=17(2)\,{{\rm{ns}}}$ with quantum-algorithmic cooling (further details in Supplementary Note 5). For Device 2, our best Rabi oscillations reach Ω = 273(6) MHz and *Q* = 18.8(3), and our best measured coherence is ${T}_{2}^{*}=74(11)\,{{\rm{ns}}}$ with quantum-algorithmic cooling – in this case, we adapted the cooling algorithm to use the transverse collinear interaction, as further described in Supplementary Note 5. While it is tempting to make conclusions about InGaAs vs GaAs QDs on this basis, it is unclear what leads to the striking difference in performance measured here – it may simply be a difference in the optical quality of the QD heterostructure rather than a difference intrinsic to InGaAs vs GaAs QDs.

## Discussion

With this, we have demonstrated full quantum control of an electron-spin qubit in Faraday geometry for both InGaAs and GaAs QDs, where the cyclicity of spin readout transitions was measured to be ${{\mathcal{C}}}=291\,(28)$ and ${{\mathcal{C}}}=471\,(50)$, respectively. In this configuration, we have showed nuclear-spin cooling to enhance the electronic inhomogeneous dephasing time ${T}_{2}^{*}$ to tens of nanoseconds, which is sufficient to resolve and address nuclear magnons towards quantum-memory operation^17^. We note that while tens of nanoseconds of ground-state ${T}_{2}^{*}$ coherence is relatively short in comparison with other solid-state platforms^59,60^, this enhanced value is two orders of magnitude longer than the radiative lifetime of a QD trion (hundreds of picoseconds even in the absence of Purcell enhancement). Combined with spin rotations, which are not limited by nuclear-spin noise (Fig. 3b), our results are thus compatible with high-fidelity spin-photon entanglement^31^. As compared with the state-of-the-art in Voigt geometry of ${T}_{2}^{*} > 600\,{{\rm{ns}}}$^49^ and *Q* = 45 ^17^ on an electron spin in a GaAs QD, we attribute the lower spin coherence and rotation fidelity to the coupling of the qubit splitting to laser intensity fluctuations via the differential Stark shift and to the increased relative importance of laser-induced relaxation in the presence of a weaker spin-flipping transition. Further improvements include stabilizing the laser intensity at the QD position to better than 0.1 % via vibration isolation and optimized power stabilization. Additionally, applying a vectorial magnetic field (i.e., a combination of Faraday and Voigt configurations) or a controlled few-percent strain to increase HH-LH mixing (on a GaAs QD^61,62^) would increase the strength of the spin-flipping transition and reduce the induced differential Stark shift, while keeping the cyclicity sufficiently high for single-shot readout. Single-shot measurements are not currently possible in our system due to a low end-to-end efficiency of less than 1 % after propagating through the microscope, optical filters, and single-photon detector. With a cyclicity of  ≈ 500, an improvement to  ~ 10% efficiency would suffice, made straightforward by photonic structures now reaching 70% end-to-end efficiency with similar QDs^12,63,64^, which combined with our method, would allow for the generation of photonic cluster states with tens to hundreds of photons.

## Methods

### Experimental setup

Device 1 (Device 2) is held at 4 K (3.4 K) inside a helium bath cryostat (closed-cycle cryostat) where a superconducting magnet produces a field *B*_*z*_ up to 8 T in the *z*-direction. An objective lens with a Numerical Aperture of 0.5 (Thorlabs C240TMD-B) collects light from Device 1, while an objective lens with a Numerical Aperture of 0.7 (Thorlabs C330TMD-B) collects light from Device 2. A resonant laser and a Raman laser are combined on a beam splitter before they are sent into the cryostat. The QD emission is then coupled back into an optical fiber before it is detected on an avalanche photodiode (APD) and time-tagged with a Swabian Time Tagger. We employ cross-polarization in the photon collection path to reject the resonant readout laser, along with spectral filtering using a transmission grating setup (30 GHz full-width-at-half-maximum) to further reject the Raman laser.

The resonant laser is linearly-polarized to enable equal excitation (and collection) of both the circularly-polarized dipoles of the trion *X*^1−^. We use an acousto-optical modulator (AOM) to pulse and actively stabilize the resonant laser power during experiments.

The Raman laser system consists of a pair of red-detuned lasers with equal intensities, derived from electro-optical modulation of a single narrow-linewidth laser. This is done using a fiber-coupled amplitude-electro-optical modulator (EOM), which is locked to its interferometric minimum, and is driven by microwave pulses generated from an arbitrary waveform generator (AWG). Due to a large *ω*_*e*_ (> 20 GHz) in Device 1 (with InGaAs QDs), we use an IQ mixer (Marki Microwave, MMIQ-0218LXPC) to upconvert the output from two channels of an AWG (Tektronix 70002B) to  ≃ 15 GHz with a microwave signal generator (Rohde and Schwarz, SMF100) as local oscillator. In the case of Device 2 (with GaAs QDs), due to the smaller *ω*_*e*_ (< 5 GHz), we directly synthesize the microwave pulses in the time domain using a single channel of an AWG (Real Digital, RFSoC 4 × 2, up to 9.85 GSPS) instead. We build upon the *qick* firmware^65^ and develop a Python library (*qeg-rfsoc*^66^) to interface with the board. To minimize the photo-refractive effect in our EOM that causes large fluctuations in the extinction ratio and drift in the EOM bias, we seed the EOM with a laser power of  ~ 10 mW. We place an AOM after the EOM to pulse and actively stabilize the intensity of the Raman laser. We then amplify the signal with an optical amplifier (Thorlabs, BOA780) and spectrally filter the amplified signal with a grating filter (full-width-at-half-maximum of 35 GHz) to remove the broad amplified spontaneous emission from the amplifier. The filtered laser output is then coupled into an optical fiber before propagating through the Raman path. A Swabian Pulse Streamer is used to trigger the AWG and Swabian Time Tagger 20 and to generate optical pulses via electrical pulses to the AOM drivers.

### Modified quantum algorithmic cooling

Figures 5a, b, 6f show the modified quantum-algorithmic cooling sequence we use to stabilize the ESR, from which we achieve an electronic ${T}_{2}^{*}=74(11)\,{{\rm{ns}}}$. In place of the original quantum-algorithmic cooling scheme^25^ (used with the InGaAs QD in Device 1), we add an extra *π*/2 pulse, whose phase is *π*/2 relative to the first *π*/2 pulse, following the sensing period of the algorithm and directly before the electro-nuclear interaction (hh) pulse. This additional pulse turns an electronic coherence along  ± *x* to  ± *z* to enable a nuclear spin flip conditioned on the electronic state, with the single-sideband process allowed by the collinear hyperfine interaction (see Fig. 5b). The cooling algorithm uses the following parameters to obtain the data of Figs. 5a, b, 6f: fixed sensing time of *t*_sense_ = 55 ns, *N* = 30, Ω_*hh*_ ≈ 47 MHz, *τ*_*hh*_ = 60 ns, and *ϕ*_sense_ = 0.875*π*. Note that *ϕ*_sense_ is not *π*/2 here, only owing to an uncompensated free precession phase. The *π*/2 − pulses are done at Ω = 125 MHz, corresponding to a duration of 2 ns. The cooling sequence is dominated by the 1.5 μ*s*-long resonant pulse to reset the spin, resulting in a total cooling duration of  ~ 55 μ*s*.

## Supplementary information

Supplementary Information

Transparent Peer Review file

## Data Availability

Data described in this paper and presented in the Supplementary Information are openly available^67^.
