# Supplementary material for: Coherent control of quantum-dot spins with cyclic optical transitions
Source: Nat Commun. 2026 Jun 24;17:7917. doi: 10.1038/s41467-026-74590-z (PMC13444142; doi:10.1038/s41467-026-74590-z)
Supplement: Supplementary file 1 [file 41467_2026_74590_MOESM1_ESM.pdf]

Supplementary Information: Coherent Control of Quantum-Dot Spins with Cyclic Optical Transitions

Zhe Xian Koong,[1,*] Urs Haeusler,[1] Jan M. Kaspari,[2] Christian Schimpf,[1] Benyam Dejen,[1] Ahmed M. Hassanen,[1,3] Daniel Graham,[1] Yusuf Karli,[1] Ailton J. Garcia Jr.,[4] Melina Peter,[4] Edmund Clarke,[5] Maxime Hugues,[6] Michał Gawełczyk,[7] Armando Rastelli,[4] Doris E. Reiter,[2] Mete Atatüre,[1,†] and Dorian A. Gangloff[1,‡]

[1]*Cavendish Laboratory, University of Cambridge, JJ Thomson Ave, Cambridge CB3 0HE, United Kingdom*
[2]*Department of Physics, TU Dortmund University, 44221 Dortmund, Germany*
[3]*Department of Engineering Science, University of Oxford, Parks Road, OX1 3PJ, United Kingdom*
[4]*Institute of Semiconductor and Solid State Physics, Johannes Kepler University, Linz, Austria*
[5]*EPSRC National Epitaxy Facility, University of Sheffield, Broad Lane, Sheffield S3 7HQ, United Kingdom*
[6]*Université Côte d'Azur, CNRS, CRHEA, rue Bernard Gregory, 06560 Valbonne, France*
[7]*Institute of Theoretical Physics, Wrocław University of Science and Technology, Wrocław, Poland*
(Dated: June 4, 2026)

Supplementary Note 1. DEVICE PROPERTIES AND SPIN T_1

Device 1 consists of self-assembled InGaAs QDs embedded in a Schottky diode structure for deterministic charge control via the applied gate voltage. The heterostructure is presented in Fig. Supplementary Fig. 1(a), taken from Refs. [1–3]. The gate voltage induces a d.c. Stark shift and tunes the emission wavelength of the emitter. This is depicted in the photoluminescence data presented in Fig. Supplementary Fig. 1(b) where we observe the presence and the splitting of the emission lines from the neutral (X^0) and charged excitons (X^{1-}, X^{1+} and X^{2-}) at $B_z = 3.5\,\mathrm{T}$ when exciting the QD with an above-band laser at 780 nm. There is a 35-nm tunnel barrier between the n-doped layer and the QDs, and a capping layer above the QD layer to prevent charge leakage. A planar cavity consisting of 20 pairs of distributed Bragg reflectors (DBR) and a super hemispherical cubic zirconia solid immersion lens (SIL), which effectively augments the Numerical Aperture of the objective from 0.5 to 1.15 [4], are used to maximize photon out-coupling efficiency to $\sim 10\,\%$ for QDs with an emission wavelength around 970 nm.

Device 2 consists of Al-droplet-etched GaAs QDs, grown via molecular beam epitaxy, in a p-i-n diode. The heterostructure is presented in Fig. Supplementary Fig. 1(d). To increase the photon out-coupling, the heterostructure contains 40 pairs of DBR mirror. By applying a weak forward bias, we can deterministically charge the QD and induce a d.c. Stark shift to the emission wavelength. We observe clear splitting of the emission lines at $B_z = 6.5\,\mathrm{T}$ when excited with a 632 nm above-band laser. The photoluminescence emission as a function of device bias is shown in Fig. Supplementary Fig. 1(e).

We estimate the electron-spin lifetime, Γ_1^{-1} by performing time-resolved spin pumping experiments on the X^{1-} transition. By varying a delay between two resonant readout pulses, we can measure the readout counts normalized to the initialization counts as a function of delay. We extract a Γ_1^{-1} of $51(1)\,\mu s$ and $43(2)\,\mu s$ for Devices 1 and 2, respectively, by fitting an exponential function to the counts as a function of delay. These results are shown in Fig. Supplementary Fig. 1(c) and Supplementary Fig. 1(f).

We estimate the spin-initialization fidelity from measuring the time-resolved photon arrival histogram during spin pumping, as a function of the QD-laser detuning, as set by the sample voltage bias via the DC Stark shift. Fig. Supplementary Fig. 2(a) shows an example of the photon arrival histogram as a function of sample bias for the QD in Device 1. We observe a shorter spin-pumping time when it is tuned to resonance. Examples of the photon arrival histograms at device biases corresponding to on and off-resonance on the QDs in Device 1 and Device 2, at powers well above saturation, are shown in Fig. Supplementary Fig. 2(b) and Supplementary Fig. 2(c), respectively. For Device 1 (Fig. Supplementary Fig. 2(b)), by subtracting the photon arrival histogram off-resonance from that on resonance, we can attribute the steady-state counts predominantly to the laser reflection background. For Device 2 (Fig. Supplementary Fig. 2(c)), the steady-state counts can be attributed to repumping by the readout laser due to the small ground-state splitting and high optical power (with Rabi frequency comparable to the ground-state splitting). This is highlighted by the higher off-resonant steady-state counts (grey curve) compared to that on resonance in Fig. Supplementary Fig. 2(c). For measurements on the GaAs QD in Device 2 presented in the main text in Figs. 2-6, we thus use an optical power below saturation ($\sim 0.5 P_{\mathrm{sat}}$) and a higher magnetic field (6.5 T) for spin initialization and readout to circumvent this error. This is shown in Figure Supplementary Fig. 2(d), where the steady-state counts at a bias corresponding to being far off-resonance, where they are dominated

* Corresponding author: zxk22@cam.ac.uk
† Corresponding author: ma424@cam.ac.uk
‡ Corresponding author: dag50@cam.ac.uk

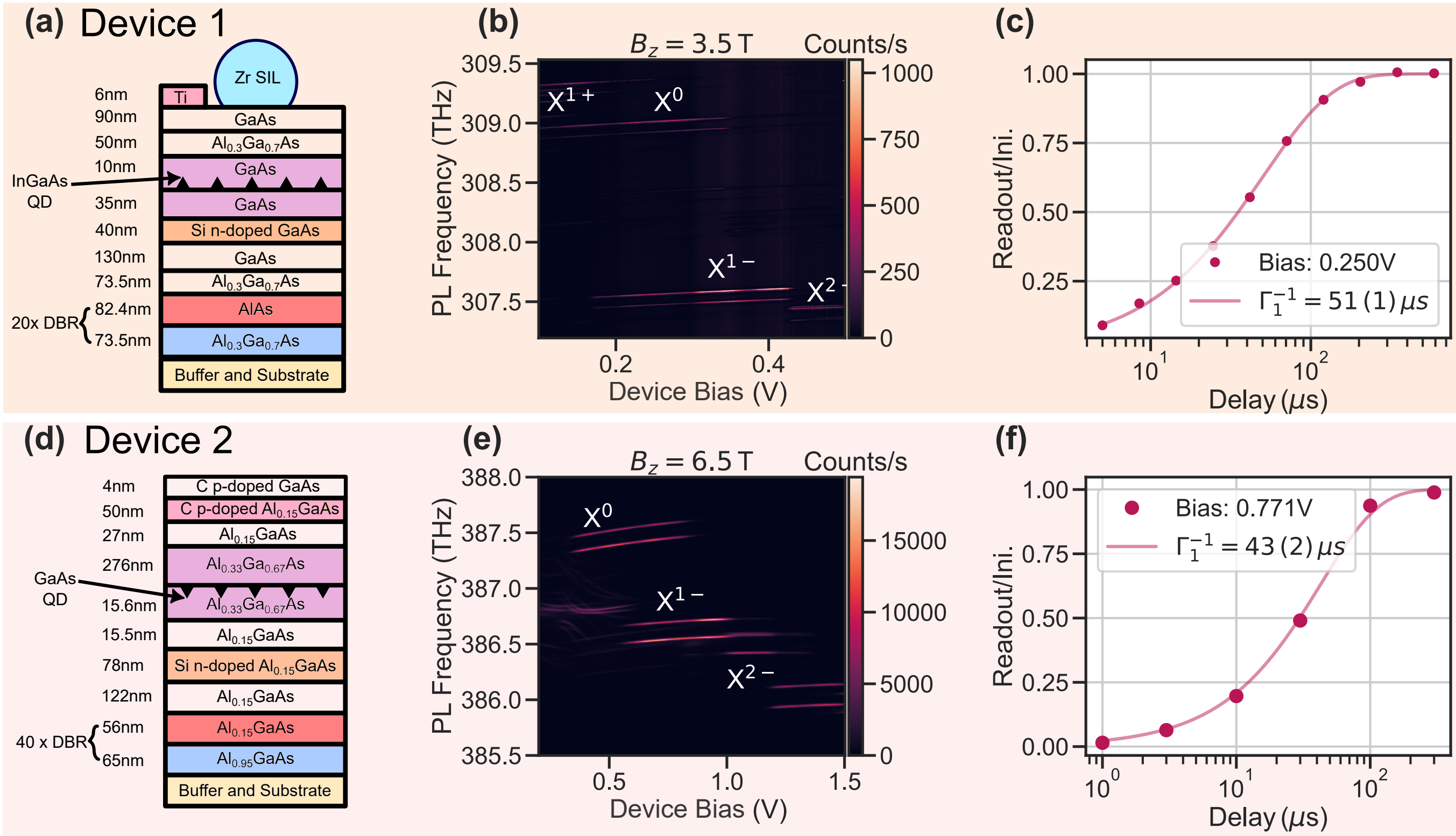

Supplementary Fig. 1. **(a)-(c)** (**(d)-(f)**) are measurements taken on Device 1 (Device 2). (a) and (d) are device heterostructures. (b) and (e) are photoluminescence emission frequency as a function of Device bias for above-band excitation. (c) and (f) are electron-spin relaxation experiments consisting of a resonant spin initialization (ini.) pulse, a variable delay, and a resonant spin readout pulse. The data points are the ratio of readout to initialization counts. The solid curves are fits to the data with an exponential function whose characteristic time is Γ_1^{-1}, as reported in the legend. Error bars indicate one standard error on fitted parameters.

by laser background, match the steady-state counts at a bias corresponding to resonance.

For the settings that lead to a steady-state limited by laser background, the spin pumping rate $\gamma_{\rm SP}$ can be extracted from the fits (Fig. Supplementary Fig. 2(b) and (d)), and one can calculate an upper bound to the spin-pumping fidelity [5], limited by Γ_1 and the spin pumping rate $\gamma_{\rm SP}$, of $F_{\rm sp}^{\rm upper} = (1+4\Gamma_1/\gamma_{\rm SP})^{-1} = 98\%$ (97%) for QDs in Device 1 (Device 2).

Supplementary Note 2. HEAVY-HOLE LIGHT-HOLE MIXING AND RAMAN SELECTION RULES

A. Hole subband mixing

We follow the HH-LH mixing treatment in the presence of strain from Ref. [6], which includes both direct first-order coupling between subbands and second-order terms involving the remote spin-orbit split-off band. In addition, we consider the spatial dependence of band admixtures. The relevant terms for understanding the spin-flipping transitions involve the valence subbands $|J, J_z\rangle$, where $J \in \{3/2, 1/2\}$ and J_z is the projection along the growth axis. We ignore any direct contributions of the split-off band $J = 1/2$ to the eigenstates, which are negligible in comparison with mixing within the $J = 3/2$ manifold, where $J_z = \pm 1/2$ and $J_z = \pm 3/2$ are the Light-Hole (LH) and Heavy-Hole (HH) valence subbands, respectively. However, we retain second-order virtual coupling via the split-off band, which yields additional mixing contributions in the $J = 3/2$ manifold.

Note that (solely) in Supplementary Note 2 A, we work in the *valence electron* picture. When such a valence-electron state is removed, the corresponding *hole* spinor used elsewhere in the manuscript is defined as its time-reversed spinor: $|i\rangle_{\rm h} = \mathcal{T}\,|i\rangle$, where $\mathcal{T}\,|J, J_z\rangle = (-1)^{J-J_z}\,|J, -J_z\rangle$.

In a nanostructure, electron states are generally spatially-dependent superpositions of bands,

$$|\psi(\vec{r})\rangle = \sum_{\mu} \psi_\mu(\vec{r})\,|\mu\rangle\,, \qquad \text{(S1)}$$

where μ labels the bands [that also include the Conduction (C) band with $J = 1/2$ in addition to the mentioned valence bands] and ψ_μ are the distinct spatial envelopes for each band. We will further expand the envelopes in some basis of orbital states (e.g., 3D harmonic oscillator

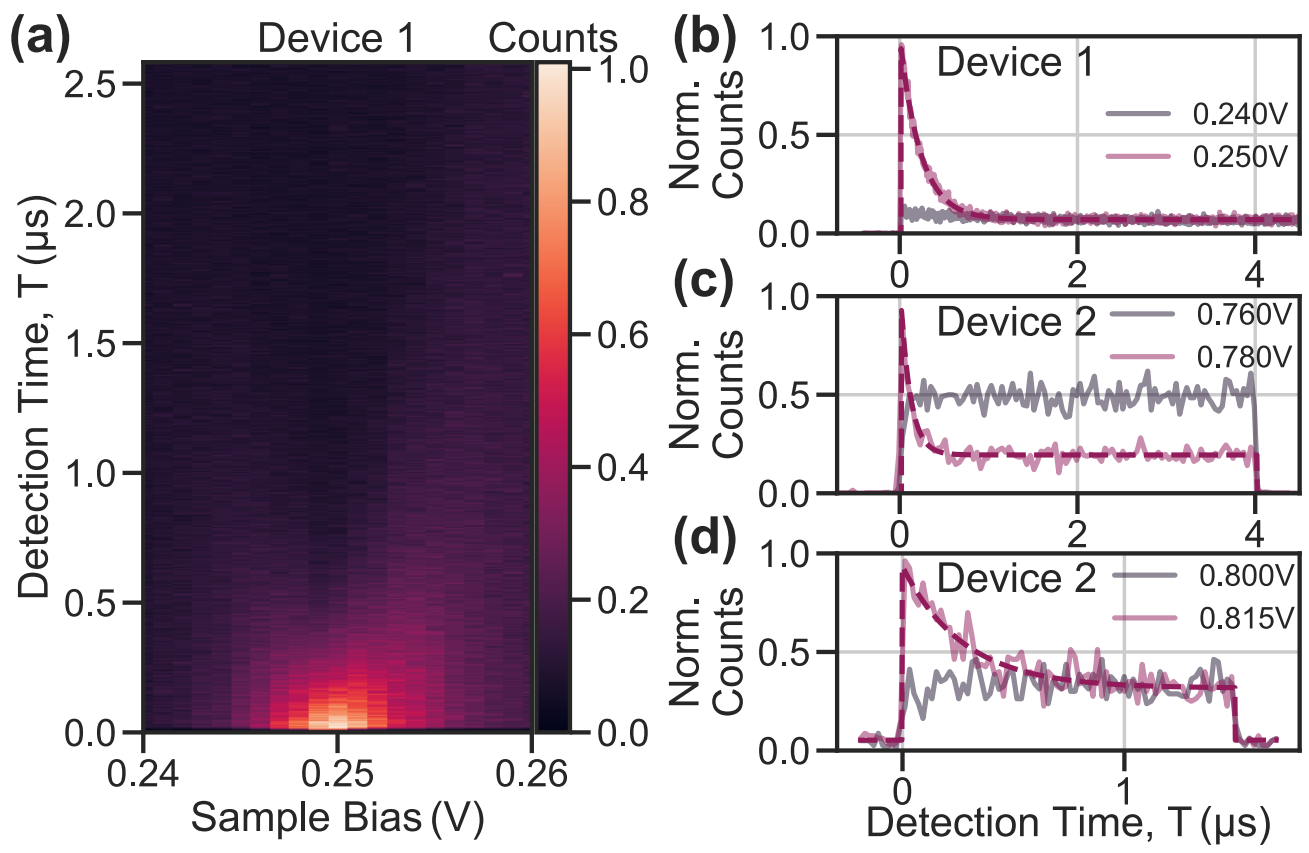

Supplementary Fig. 2. **(a)** Photon arrival histogram, from the X^{1-} emission for the QD in Device 1 at $B_z = 3.5\,\mathrm{T}$, as a function of sample bias, for the initialization pulse. **(b)** (**(c)**) Example histogram when the QD is bias-tuned to be resonant and off-resonant with the excitation laser in Device 1 (Device 2) with laser power of $\sim 16\,P_{\mathrm{sat}}$ ($\sim 6\,P_{\mathrm{sat}}$), measured at $B_z = 3.5\,\mathrm{T}$. The spin pumping time, obtained from the exponential fit on resonance at 0.250 V (0.780 V) is $\gamma_{\mathrm{SP}}^{-1} = 234\,(2)\,\mathrm{ns}$ ($\gamma_{\mathrm{SP}}^{-1} = 111\,(17)\,\mathrm{ns}$). These data are from the same data set that generates the figures in Fig. 1(d) and 1(e). **(d)** Example histogram when the QD is bias-tuned to be resonant and off-resonant with the excitation laser in Device 2 with a laser power of $\sim 0.5\,P_{\mathrm{sat}}$, measured at $B_z = 6.5\,\mathrm{T}$. The spin pumping time, obtained from the exponential fit on resonance at 0.815 V is $\gamma_{\mathrm{SP}}^{-1} = 276\,(32)\,\mathrm{ns}$, with the steady-state counts limited only by laser background.

orbital states),

$$\psi_\mu(\vec{r}) = \sum_i a_i^{(\mu)} \phi_i^{(\mu)}(\vec{r}). \tag{S2}$$

For C-band electron states, we can safely neglect any band mixing and assume simple

$$|\uparrow/\downarrow\rangle = \phi_0^{(\uparrow/\downarrow)} \left|\frac{1}{2}, \pm\frac{1}{2}\right\rangle \tag{S3}$$

states modulated by spatially even ground-state envelopes. In contrast, band mixing in the valence band is generally strongly position-dependent (via different envelopes for different subbands), so it is impossible to represent a valence electron state as a position-independent ket.

Let H_v be the 6x6 valence-band Hamiltonian expressed in the $|J, J_z\rangle$ basis. Represented in our product basis of confined states, $\{\phi_i\,|\mu\rangle\}$, it takes the form

$$H_v = \begin{pmatrix} \langle H_v\rangle_{00} & \langle H_v\rangle_{01} & \cdots \\ \langle H_v\rangle_{10} & \langle H_v\rangle_{11} & \cdots \\ \vdots & \vdots & \ddots \end{pmatrix}, \tag{S4}$$

where $\langle O\rangle_{ij} = \int \mathrm{d}^3\vec{r}\,\phi_i^*(\vec{r})\,O\,\phi_j(\vec{r})$ and thus $\langle H_v\rangle_{ij}$ are 6×6 blocks for each combination of the envelope basis functions, describing the coupling between the envelope sectors i and j. Then, we look for LH contributions to the predominantly HH hole ground state that are generated by the off-diagonal elements of this Hamiltonian between $\phi_0(\vec{r})\,|3/2, \pm 3/2\rangle$ and some $\phi_i(\vec{r})\,|3/2, \pm 1/2\rangle$ for any i. Thus, a given total LH admixture has an envelope ψ_μ composed of many envelope basis functions ϕ_i of different symmetry.

For that reason, it is impossible to provide a simple representation of the hole states, as band mixing is generally position-dependent. However, for optical transitions, we are only interested in the transition dipole moment matrix elements taken between conduction and valence (hole) states, $\langle c|\vec{d}|v\rangle$, where $c = \uparrow, \downarrow$ and v stands for the valence (time-reversed hole) eigenstate. Under our assumption on the conduction states, this becomes

$$\begin{aligned} \vec{d}_{cv} &\simeq \sum_\mu \int \mathrm{d}^3\vec{r}\,\phi_0^*(\vec{r})\,\psi_\mu(\vec{r})\,\langle c|\vec{d}|\mu\rangle \\ &= \langle c|\,\vec{d} \sum_\mu \int \mathrm{d}^3\vec{r}\,\phi_0^*(\vec{r})\,\psi_\mu(\vec{r})\,|\mu\rangle\,, \\ &\equiv \langle c|\vec{d}|V\rangle\,, \end{aligned} \tag{S5}$$

which shows that for optical transitions only the projections of the hole mixing envelopes onto the even electron envelope matter. This allows us to write such projected, position-independent 'effective' lowest valence states associated with the lowest hole doublet, $|V\rangle = \int \mathrm{d}^3\vec{r}\,\phi_0^*(\vec{r})\;|v\rangle$ as:

$$\begin{aligned} |\Uparrow\rangle &= \alpha \left|\frac{3}{2}, +\frac{3}{2}\right\rangle + \chi \left|\frac{3}{2}, +\frac{1}{2}\right\rangle + \epsilon \left|\frac{3}{2}, -\frac{1}{2}\right\rangle, \\ |\Downarrow\rangle &= \alpha \left|\frac{3}{2}, -\frac{3}{2}\right\rangle - \chi^* \left|\frac{3}{2}, -\frac{1}{2}\right\rangle + \epsilon^* \left|\frac{3}{2}, +\frac{1}{2}\right\rangle, \end{aligned} \tag{S6}$$

where $\alpha \lesssim 1$ parametrizes the dominant HH contribution (without loss of generality, we take α to be real and thus our phase reference), and χ and ϵ to be small complex numbers parameterizing the optically-active part of the LH contribution. To relate this to the hole picture, we use the convention that the hole spinor associated with a missing valence electron is the time-reversed spinor of that electron. Thus, e.g., a missing $|\Uparrow\rangle$ electron corresponds to $|\Downarrow\rangle_{\mathrm{h}} = \mathcal{T}\,|\Uparrow\rangle$, which has formally the same spinor expansion as $|\Downarrow\rangle$. In spin-preserving transitions, the contribution of the χ term is dark, while ϵ creates a small opposite circular polarization contribution leading to ellipticity of the transition dipoles. The spin flipping transitions $\Uparrow_{\mathrm{h}} \to \downarrow$ and $\Downarrow_{\mathrm{h}} \to \uparrow$ allowed by the χ term are circularly polarized and by the ϵ term π_z polarized.

For H_v, we combine the Luttinger-Kohn valence-band Hamiltonian with the Bir-Pikus Hamiltonian accounting for strain. We then get

$$\begin{aligned} \chi &= \int \mathrm{d}^3\vec{r}\,\phi_0^*(\vec{r})\,\chi(\vec{r}), \\ \epsilon &= \int \mathrm{d}^3\vec{r}\,\phi_0^*(\vec{r})\,\epsilon(\vec{r}), \end{aligned} \tag{S7}$$

with

$$\chi(\vec{r}) = \sum_i \frac{-\sqrt{2}\Delta_{\mathrm{SO}}\langle S\rangle_{0i}^* - \sqrt{6}\langle S\rangle_{0i}\langle R\rangle_{0i}^*}{\langle\Delta_{\mathrm{lh}}\rangle_{0i}\Delta_{\mathrm{SO}}}\,\phi_i(\vec{r}),$$
$$\epsilon(\vec{r}) = \sum_i \frac{(\frac{3}{2}\langle\Delta_{\mathrm{lh}}\rangle_{0i} + \Delta_{\mathrm{SO}})\langle R\rangle_{0i}^* + \sqrt{3}(\langle S\rangle_{0i}^*)^2}{\langle\Delta_{\mathrm{lh}}\rangle_{0i}\Delta_{\mathrm{SO}}}\,\phi_i(\vec{r}), \tag{S8}$$

where

$$R(\vec{r}) = -\frac{\hbar^2}{2m_0}\sqrt{3}\left[\gamma_2(k_x^2 - k_y^2) - 2i\gamma_3 k_x k_y\right] + \frac{\sqrt{3}}{2}b\left[e_{xx}(\vec{r}) - e_{yy}(\vec{r})\right] - ide_{xy}(\vec{r}),$$
$$S(\vec{r}) = -\frac{\hbar^2}{2m_0}2\sqrt{3}\,\gamma_3(k_x - ik_y)k_z + \frac{d}{\sqrt{2}}\left[e_{zx}(\vec{r}) - ie_{yz}(\vec{r})\right] \tag{S9}$$

are the relevant off-diagonal HH-LH mixing matrix elements of H_v in the band basis, and

$$\Delta_{\mathrm{lh}}(\vec{r}) = E_{\mathrm{HH}}^{(0)} - E_{\mathrm{LH}}^{(0)} = -\frac{\hbar^2}{2m_0}2\gamma_2\left(k_z^2 - \frac{k_x^2 + k_y^2}{2}\right) + \frac{b}{2}\left[e_{xx}(\vec{r}) + e_{yy}(\vec{r}) - 2e_{zz}(\vec{r})\right], \tag{S10}$$

is the splitting between the HH and LH band edges ($E_{\mathrm{HH}}^{(0)}$ and $E_{\mathrm{LH}}^{(0)}$) in the absence of band mixing. Here, Δ_{SO} is the spin-orbit coupling constant, e_{ij}, $i,j \in \{x,y,z\}$, is the strain tensor, m_0 is the free-electron mass, k_i ($i \in \{x,y,z\}$) are the components of the crystal-momentum operator, and γ_2, γ_3 are the Luttinger parameters characterizing the valence-band effective masses.

Let us first analyze the structure of terms in Eqs. (S8) and (S9), focusing on differences between GaAs and InAs QDs and temporarily neglecting the position dependence. Hole subband splitting, Δ_{lh}, has contributions due to confinement and biaxial strain. For larger and almost unstrained GaAs QDs, both contributions are typically small compared to spin-orbit interaction, hence, in terms of magnitude, $\Delta_{\mathrm{lh}} \ll \Delta_{\mathrm{SO}}$, $|SR^*| \ll |\Delta_{\mathrm{SO}}S^*|$ and $|S^2| \ll |R|$. As a result, we can initially expect $\chi \approx -\sqrt{2}S^*/\Delta_{\mathrm{lh}}$ and $\epsilon \approx R^*/\Delta_{\mathrm{lh}}$. For InGaAs QDs, however, the confinement is stronger, and also no strain contribution can be ignored, as strain energy scales can be comparable to spin-orbit coupling; as a result, no such simplification can generally be made to these terms.

On top of that, symmetry of the envelopes comes into play via parity selection rules for the electron and hole envelopes (momentum conservation relaxed by confinement). From Eq. (S9), we learn that both R and S have kinetic and strain contributions. The kinetic part of R is even and therefore can only produce even contributions to LH admixture envelopes. Additionally, it requires in-plane anisotropy of the QD confinement potential. Thus, it is typically present in InAs QDs, where shear strain and piezoelectric field break the rotational symmetry of confinement even in otherwise symmetric QDs, leading to significant ellipticity of allowed transitions and significant π_z polarized contribution to forbidden transitions. Apart from the QD shape, engineered external fields that deform the hole wave function could be used to tune this effect. On the other hand, in less strained and close to rotationally symmetric GaAs QDs, this contribution can be negligible. The strain part of R relies on the in-plane shear strain, which is a sign-changing field around the dot and predominantly couples the ground state to the admixture envelopes of odd in-plane symmetry. As a result, GaAs QDs with low biaxial strain and resultant low HH-LH subband splitting can still have a significant ϵ admixture, which is, however, odd and thus does not contribute to the allowed optical transitions. In InAs QDs, this strain contribution to R is present but does not affect the allowed transitions.

Inspecting S, we can see that its kinetic part is odd along the growth axis, since it involves k_z. Any admixture this part could produce would therefore require an envelope excited along that direction, which strongly suppresses this contribution in both types of dots, especially in flat InAs QDs. The important part thus comes from the shear strains in the vertical planes. These, again, are odd-like functions and thus couple to odd envelopes. However, S enters the equation for ϵ squared and in the one for χ multiplied by R in one of the terms. The term SR is therefore most likely responsible for the spatially even and bright contribution to the χ admixture in both types of QDs. It also produces a minor contribution to the bright part of ϵ. Thus, shear-strain engineering seems to be the route to tune this admixture, while biaxial strain compensating for the built-in HH-LH splitting can enhance both types of admixtures, especially in GaAs QDs.

Up to this point, we neglected the impact of the magnetic field on HH-LH subband mixing. The difference in Faraday-geometry g-factors for HH and LH valence bands, $g_{\mathrm{HH}} = 6\kappa + 27q/2$, $g_{\mathrm{LH}} = 2\kappa + q/2$ (with $\kappa = 1.28$, $q = 0.04$ in GaAs [7]), leads to different values of Δ_{lh} when different spin configurations are considered, with the Zeeman contribution being

$$\Delta^{(Z)}_{\mathrm{lh};\Uparrow/\Downarrow,\uparrow/\downarrow} = \frac{1}{2}\left(\pm g_{\mathrm{LH}} \mp g_{\mathrm{HH}}\right)\mu_{\mathrm{B}}B_z, \tag{S11}$$

and the $\pm$ symbols referring independently to the orientations of the LH- and HH-subband spins. Note that signs in Eq. (S11) need to be flipped when hole states are considered. In InGaAs QDs, this can be neglected in view of large splitting due to strain, but in GaAs QDs, these Zeeman contributions can be significant and lead to unequal LH admixtures to the states $|\Uparrow\rangle$ and $|\Downarrow\rangle$ in

Eq. (S6). In particular, unequal values of χ lead to the difference in the imbalances η on the two Λ subsystems.

B. Effect on Raman Transitions

For a general Raman laser polarization (where, as in our experiment, both Raman fields are the same polarization), we have two lambda systems, corresponding to two trion intermediate states, operating in parallel. The first in which the trion state with $\Downarrow$ links a spin-conserving transition in absorption, $(\Omega_{\downarrow}^{\mathrm{sc}})^*$, and a spin-flipping transition in emission, $\Omega_{\uparrow}^{\mathrm{sp}}$. The second in which the trion state with $\Uparrow$ links a spin-flipping transition in absorption, $(\Omega_{\downarrow}^{\mathrm{sp}})^*$, and a spin-conserving transition in emission, $\Omega_{\uparrow}^{\mathrm{sc}}$. The total Raman coupling between the two ground states is then:

$$\Omega = \frac{(\Omega_{\downarrow}^{\mathrm{sc}})^* \Omega_{\uparrow}^{\mathrm{sp}}}{2\Delta} + \frac{(\Omega_{\downarrow}^{\mathrm{sp}})^* \Omega_{\uparrow}^{\mathrm{sc}}}{2(\Delta + \omega_h)} \tag{S12}$$

Let us first consider Raman fields with a polarization in the (x, y) plane, for which $|\Omega_{\sigma_-}|^2$ and $|\Omega_{\sigma_+}|^2$ are proportional to laser intensity in the σ_- and σ_+ modes, respectively. In this situation, only the χ term LH mixing (Eq. S6) is relevant, as it is the one that allows in-plane polarized spin-flipping transitions. The coupling between the ground states becomes:

$$\Omega = \frac{-\chi_{\Downarrow}^* |\Omega_{\sigma_-}|^2}{2\Delta} + \frac{\chi_{\Uparrow}^* |\Omega_{\sigma_+}|^2}{2(\Delta + \omega_h)}, \tag{S13}$$

where we made explicit the possible difference of χ in the magnetic field. We see immediately that for $|\Omega_{\sigma_-}|^2 = |\Omega_{\sigma_+}|^2$, i.e. linearly polarized light, and in the limit of $B_z \to 0$ ($\omega_h \to 0, \chi_{\Downarrow} = \chi_{\Uparrow}$), the two lambda systems destructively interfere and $\Omega \to 0$. Including the field, we still deal with $\chi_{\Downarrow} \approx \chi_{\Uparrow}$ for InGaAs QDs, and the significant ω_h makes the coupling nonvanishing. In contrast, we already deduced $\chi_{\Uparrow} > \chi_{\Downarrow}$ for GaAs QDs, which compensates for ω_h in the denominator, keeping Ω close to vanishing, as we see for a GaAs QD (Fig. 2 and Supplementary Note 3).

If we introduce a coupling of the Raman fields to π_z – which is possible when focusing our laser through an objective with large numerical aperture taking us away from the paraxial limit – and parametrize it as a fraction η of a linearly polarized field Ω_π in-plane, then the coupling between the ground states becomes:

$$\Omega = \frac{\Omega_{\sigma_-}^* (-\chi^* \Omega_{\sigma_-} + \epsilon^* \eta \Omega_\pi)}{2\Delta} + \frac{\Omega_{\sigma_+} (\chi \Omega_{\sigma_+}^* + \epsilon^* \eta \Omega_\pi^*)}{2(\Delta + \omega_h)}. \tag{S14}$$

Here, a deviation from pure circular polarization would activate the π_z contribution and could enhance the Raman coupling. This would require a significant value of the ϵ LH mixing term arising, for example, from in-plane asymmetry of the confinement, and in-plane shear strains. InGaAs QDs exhibit both, and this mechanism could thus explain why the optimal Raman coupling for these QDs is elliptical, as seen in Supplementary Note 5. The solid immersion lens present in Device 1 further increases the numerical aperture and could enhance this effect.

Supplementary Note 3. DEVICE 2 RAMAN POLARIZATION MAPS SUPPLEMENTARY DATA

The Raman fields used in our experiment consist of a pair of red-detuned lasers with equal intensities, derived from electro-optical modulation of a single narrow-linewidth laser. Fig. Supplementary Fig. 3(a) shows the experimental setup of the Raman laser path where we control the polarization of the Raman laser via a pair of motorized half-wave (HWP) and quarter-wave (QWP) plates. To calibrate the angles of the HWP and the QWP, we place a polarizing beam splitter (PBS) after the two wave plates and by iteratively changing the angle of both waveplates to minimize the laser light at the reflection port of the PBS, we identify the angles that correspond to the linear horizontal polarization (i.e. (HWP, QWP) = (0, 0)).

To find the optimal optical polarization, we take the ESR spectrum, by scanning the microwave frequency applied to the electro-optical modulator and detecting the readout counts, as a function of the polarization of the Raman laser pair. We employ the following pulse sequence: nuclear-spin cooling (Rabi cooling), spin initialization with a resonant pulse, probing with a Raman drive of a fixed pulse duration of 13 ns, and spin readout with a resonant pulse. We keep the laser power fixed and the cooling and probe frequencies equal to each other, $\omega_{\mathrm{cool}} = \omega$, for all of the scans. We first fix the angle of the HWP to 0° while iteratively varying the angle of the QWP. We observe a variation in the readout counts, along with a shift in the resonance as we vary the QWP angle. Fig. Supplementary Fig. 3(b) shows the two extreme cases, where we observe a maximal negative shift and a maximal positive shift from the bare electron splitting of $\omega_e^0 \approx 2.6$ GHz at (HWP, QWP)=(0°, 40°) and (HWP, QWP)=(0°, 110°), respectively. These polarization settings correspond to the dipoles of the two spin-conserving transitions, σ_- and σ_+, respectively, which are also identified from polarization-resolved photoluminescence measurements.

Scanning both the HWP and QWP angles while recording the readout counts confirms the dependence on the polarization of the shift in the ESR resonance. The bright regions in Fig. Supplementary Fig. 3(c) and Fig. Supplementary Fig. 3(d) denote the mean readout signal within the two frequency ranges where we found the ESR resonances in Fig. Supplementary Fig. 3(c) (1.9-2.8 GHz and 3.5-4.5 GHz, respectively). We

find a striking resemblance in the results depicted in Fig. Supplementary Fig. 3(c) with the variation in the degree of circular polarization, quantified by the Stokes parameter S_3, as a function of HWP and QWP angles, as shown in Fig. Supplementary Fig. 3(e). We observe a maximum red-shifted (blue-shifted) signal in the region where $S_3 = 1$ ($S_3 = -1$), corresponding to the σ_- (σ_+) polarization. These results confirm that, for Device 2, the optimal polarization for the Raman fields is the polarization aligned to the dipole of the X^{1-} transition.

Supplementary Note 4. INTENSITY NOISE MODEL AND Q

To quantify the effect of laser intensity noise on the Rabi oscillation quality factor, we systematically introduce intensity modulation on the Raman laser by modulating the voltage applied to the acousto-optical modulator (AOM) used for laser intensity stabilization. We do so by combining the output of a white noise source (derived from an arbitrary waveform generator Rigol DG832) with the output of our PID controller with a power splitter before sending them to the analog modulation port of the AOM driver. This is to ensure we have a constant noise power spectral density (up to the bandwidth of the 35 MHz waveform generator) and can explore the effect of noise from low to high frequency simultaneously. A sample-and-hold protocol is implemented at the PID controller during the experiment to ensure minimal feedback from the PID controller on the external noise. By calibrating the response of the AOM on the laser power, we can convert the peak-to-peak modulation voltage to fractional (rms) intensity fluctuation $d\mathcal{I}/\mathcal{I}$. Fig. Supplementary Fig. 4(a,c) and Supplementary Fig. 4(b,d) show the readout counts as a function of pulse duration τ at laser noise values of $d\mathcal{I}/\mathcal{I}$ of 0.00 and 0.04, respectively. Fig. Supplementary Fig. 4(a, b) (c, d) are taken at $\Omega = 60$ MHz ($\Omega = 225$ MHz). The measurements are done with the same cooling Rabi frequency, $\Omega_{\text{cool}} \approx 15$ MHz as in Fig. 3. The added intensity noise clearly degrades the quality of Rabi oscillations.

To understand and fit these dynamics, we use a two-level model calculated in *Qutip* [8, 9]. We run a master equation solver, which includes collapse operators describing the decoherence mechanisms of spin relaxation Γ_1 (fixed with values taken from the result in Fig. 6(e), i.e. $\Gamma_1 = \alpha\,\Omega, \alpha = 0.0048(3)$) and spin dephasing Γ_2 (free parameter). We also include the effect of slow detuning noise (T_2^* fixed to the Ramsey measurement value of Fig. 4(b)) by repeating the master equation simulation for a set of detunings, and performing an average of the output of the simulation weighted with a normal distribution whose standard deviation σ is defined by both the laser intensity fluctuation $d\mathcal{I}/\mathcal{I}$ and the nuclear Overhauser noise σ_{OH} i.e.

$$\sigma_{laser} = d[\Delta\delta] = (\Delta\delta/\Omega)\, d\Omega = (\Delta\delta/\Omega)\, \Omega\, d\mathcal{I}/\mathcal{I}, \quad \text{(S15)}$$

$$\sigma_{OH} = \sqrt{2}/T_2^*, \quad \text{(S16)}$$

$$\sigma = \sqrt{\sigma_{laser}^2 + \sigma_{OH}^2}. \quad \text{(S17)}$$

To simplify the fits over a range of $d\mathcal{I}/\mathcal{I}$ values, we first fit the measurement data (Rabi flops) at $d\mathcal{I}/\mathcal{I} = 0$ for each dataset to extract Ω (in the absence of any external laser-induced detuning noise). We find an average spin dephasing rate of $\Gamma_2/2\pi = 4.2(4)$ MHz for all 3 measurement settings. We fix Γ_2 to this value, and for each value of Ω, we then run the simulations as a function of $d\mathcal{I}/\mathcal{I}$. The ratio of differential Stark shift to the probe Rabi frequency is fixed to the mean experimental value $\Delta\delta/\Omega = 7.4$ found in Fig. 2(f).

Solid curves in Fig. Supplementary Fig. 4(a-d) are fits to our two-level model described above, allowing us to extract the Rabi quality factor from the π-pulse contrast f_π via $Q = -1/\ln(2f_\pi - 1)$, obtained from the fitted density matrix, showing a clear decrease in Q as $d\mathcal{I}/\mathcal{I}$ increases. By varying the magnitude of $d\mathcal{I}/\mathcal{I}$ in our simulation, we obtain the full set of theoretical predictions, shown as solid curves in Fig. Supplementary Fig. 4(d), for four values of Ω. These are consistent with the experimental data (also shown in Fig. 3), and thus indicate that the drop in Q at increasing $d\mathcal{I}/\mathcal{I}$ can be explained by the coupling of intensity noise to the differential Stark effect.

Supplementary Note 5. INGAAS SUPPLEMENTARY DATA

Fig. Supplementary Fig. 5(a) shows the narrowing of the ESR resonance with Raman (top panel) and quantum-algorithmic cooling (middle panel) at $\Omega_{\text{cool}} = \Omega \approx 30$ MHz, with a probe pulse duration set to the π-pulse time of $\tau = 17$ ns. A 2.5 μs long resonant pulse is used for initialization and the readout pulses. For quantum-algorithmic cooling, we optimize the cooling parameters to be $\tau_{hh} = 105$ ns, t_{sense} chirped from 10 ns to 200 ns in $N = 30$ steps, amounting to a duration of $\sim 85\,\mu s$. We achieve an ESR linewidth (at full-width-at-half-maximum) of 261(23) MHz and 53(6) MHz for Raman and quantum-algorithmic cooling, respectively.

We also observe the effect of the differential Stark shift on the ESR resonance, shown in Fig. Supplementary Fig. 5(b). We take ESR spectra as a function of Ω, and observe a negative shift in the ESR resonance as a function of Ω. These data yield $\Delta\delta/\Omega = -65(2)$ from a linear fit, and a corresponding Rabi frequency imbalance of $\eta = 129(5)$. We can then vary the polarization of the Raman laser pair to optimize the readout signal integrated over the ESR frequency range 25-35 GHz (i.e. ± 5 GHz around the bare ESR resonance of $\omega_e^0 = 30$ GHz), as shown in Fig. Supplementary Fig. 5(c). In contrast to our

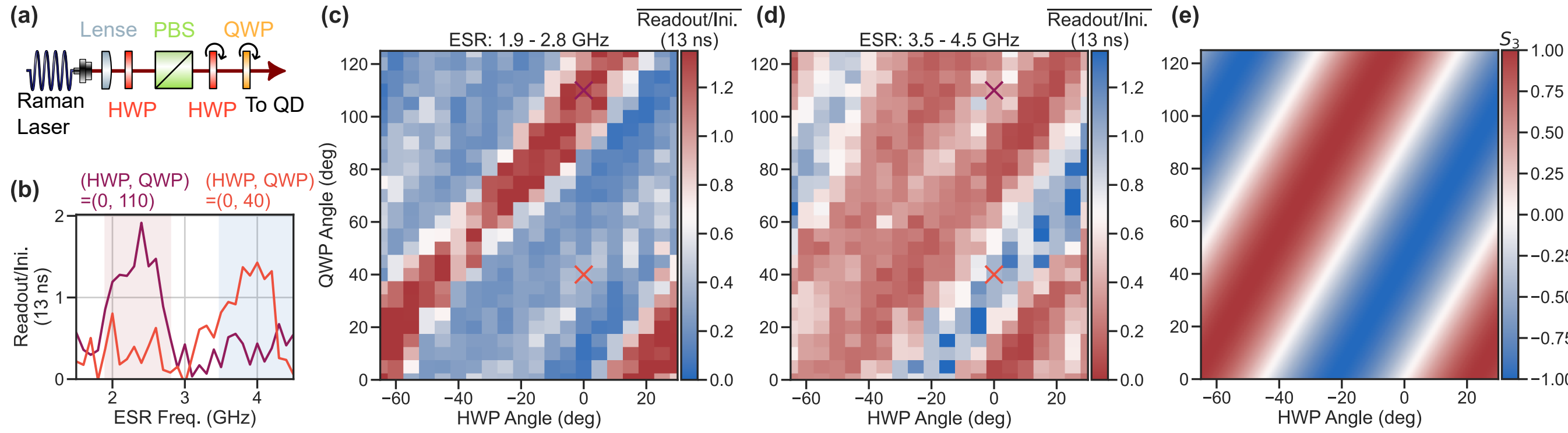

Supplementary Fig. 3. **Raman laser polarization control.** All measurements are taken on a GaAs QD in Device 2. **(a)** Schematic of the optical path for the Raman laser, highlighting the components used for polarization control. PBS: polarizing beam splitter, HWP: half-wave plate, and QWP: quarter-wave plate. **(b)** ESR spectrum at (HWP, QWP) = (0, 40°) and (HWP, QWP) = (0, 110°) showing a shift in the ESR resonance from the bare electron-spin splitting at $B_z = 6.5\ T$ of $\omega_e^0 \approx 2.6\,$GHz. **(c) (d)** Mean readout counts (normalized to the initialization counts) as a function of HWP and QWP angles in the ESR frequency range between 1.9 and 2.8 GHz (3.5 and 4.5 GHz). **(e)** Degree of circular polarization, quantified by the S_3 Stokes parameter as a function of HWP and QWP angles. $S_3 = 1(-1)$ yields $\sigma_-(\sigma_+)$ polarization.

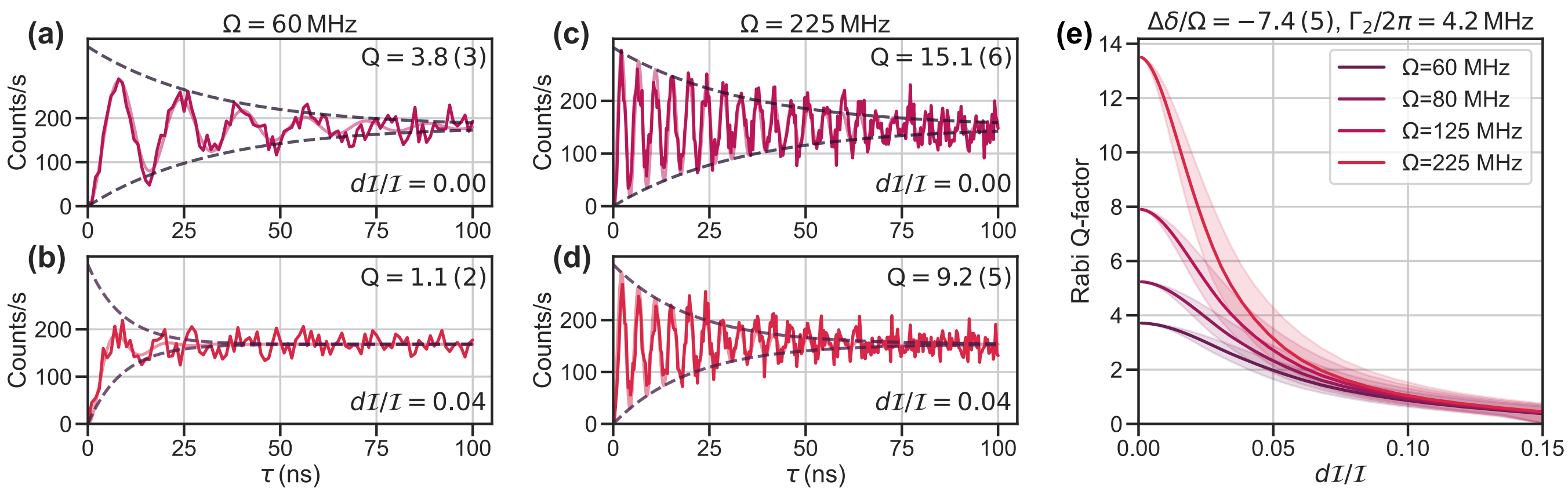

Supplementary Fig. 4. **Influence of laser intensity noise on the quality factor Q of Rabi oscillations.** All measurements are taken on a GaAs QD in Device 2. **(a, b)** Same measurement sequence as in Fig. 3. Readout counts as a function of pulse duration τ following Raman cooling ($\Omega_{\text{cool}} \approx 15\,$MHz) at fractional intensity fluctuation, $d\mathcal{I}/\mathcal{I}$ of 0.00 (**(a)**) and 0.04 (**(b)**) at the same probe Rabi frequency of $\Omega \approx 60\,$MHz. Solid curves are fits to the data using a two-level master equation model from which we obtain Q. **(c, d)** Same as (a, b) but at $\Omega \approx 225\,$MHz. **(e)** Simulated variation in Q as a function of $d\mathcal{I}/\mathcal{I}$ at various probe Rabi frequencies Ω. Here, $\Delta\delta/\Omega = 7.4(5)$ and $\Gamma_2/2\pi = 4.2\,$MHz. The shaded regions indicate three standard error on $\Delta\delta/\Omega$.

.

measurement on a GaAs QD (Fig. 2) where we found maximum readout signal with pure circular polarization, we find here that a degree of diagonal polarization $\pi_X + \pi_Y$ results in the highest readout signal. This polarization setting is used for the electron-spin control measurements shown in Fig. Supplementary Fig. 5(a-b) and Fig. 6(a-c).

Supplementary Note 6. TRION LIFETIME

Due to hardware limitations at the time of the experiments on Devices 1 and 2, we did not measure the trion lifetime of the QDs in these devices. However, since Device 1 is the same as the one used in Refs. [1, 11], we estimate the lifetime from statistics acquired in these references to be 696 (65) ps, based on the measured values of the lifetime, 584 (10) ps, 692 (5) ps, 735 (10) ps, 727 (10) ps and 742 (10) ps with QDs emitting at ~ 970 nm.

For Device 2, we estimate the trion lifetime of the QD used in the manuscript from measuring the trion lifetime of QDs from the same wafer (JKU Linz, SA1093). Using a pico-second laser (Coherent MIRA 900-D) as an excitation source and a fast detector (MPD, PD-020-CTE-FC) as the single-photon detector, we measure the trion lifetime from the QDs, one via resonance fluorescence, and two via longitudinal acoustic

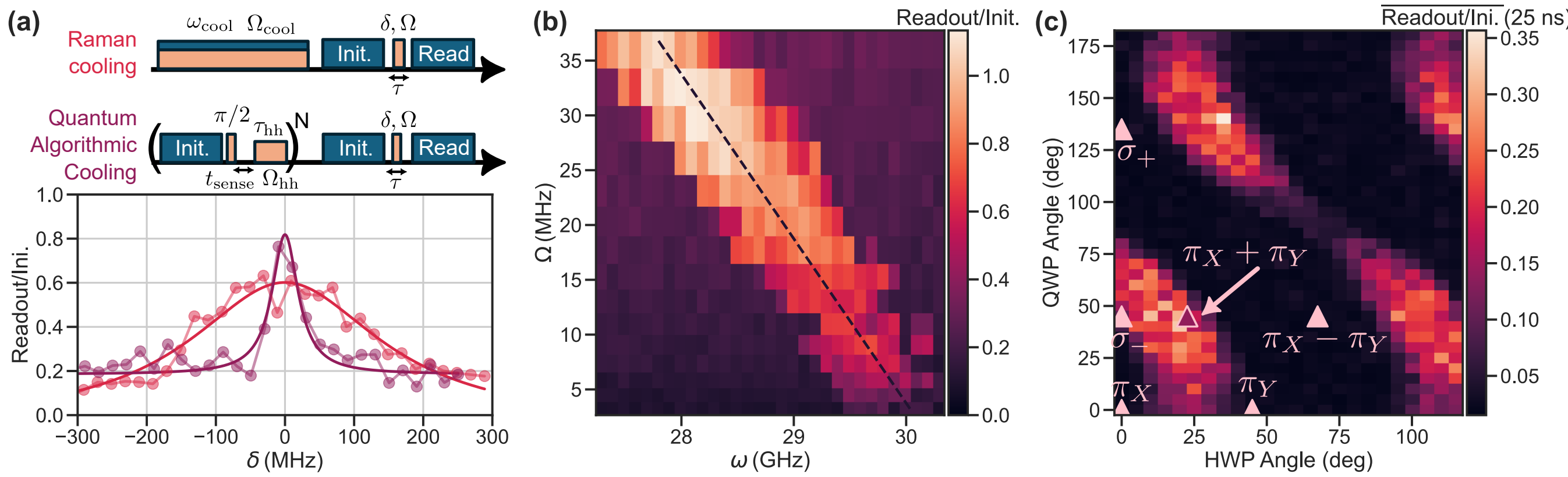

Supplementary Fig. 5. **Raman control and nuclear-spin cooling on an InGaAs QD in Faraday configuration.** All measurements are taken on an InGaAs QD in Device 1. **(a)** ESR spectroscopy. Pulse sequences (top panel) show a cooling period, with either 50 μs of Raman or 85 μs of quantum-algorithmic cooling, followed by a probe period consisting of spin initialization (2.5 μs), ESR drive, and spin readout (2.5 μs). The duration ratio of probe to cooling is $\approx$ 10 %. The bottom panel shows the resulting data with $\Omega \sim 30\,$MHz. Raman and quantum-algorithmic cooling are shown as red and purple data, respectively. A Gaussian fit (solid curves) yields linewidth (full-width-at-half-maximum) of 261(23) MHz and 53(6) MHz, respectively. At detuning δ well beyond the ESR feature, the constant value of the readout highlights the effect of spontaneous spin-flips due to optically-induced relaxation processes [10]. **(b)** Probe readout under Rabi cooling as a function of ESR drive frequency and Rabi frequency. The dashed line highlights a linear negative shift in the ESR resonance from the bare electron splitting of $\omega_e^0 \approx 30\,$GHz. **(c)** Maximum readout counts in an ESR spectrum at a fixed Raman laser power (corresponding to $\Omega \sim 20\,$MHz), under Rabi cooling, as a function of the polarization of the Raman laser, as controlled by rotating half-wave-plate (HWP) and quarter-wave-plate (QWP) angles (referenced to the linearly horizontal polarization π_X). Polarizations of interest are highlighted with triangles.

phonon-assisted excitation. Ref. [12] shows that longitudinal phonon-assisted excitation results in a similar lifetime compared to resonance fluorescence, allowing us to spectrally-separate single-photons from the stronger, broad pulse laser. We measure the photon arrival histogram, synchronized to the trigger from the pulse laser, and fit an exponential decay, convoluted with the instrument response function, which we use to extract the lifetime. We obtain a lifetime of 207 (8) ps, 254(5) ps, and 245 (8) ps for QDs emitting at 770.5 nm, 772.2 nm, and 775.5 nm, respectively. The latter two QDs are measured via longitudinal phonon-assisted excitation. An example of the lifetime data is depicted in the Fig. Supplementary Fig. 6, highlighting the fits (solid curve), the instrument-response-function (dashed curve) and the experimental data (circle dots). We obtain an estimate of 235 (25) ps for the trion lifetime, from these three measured values.

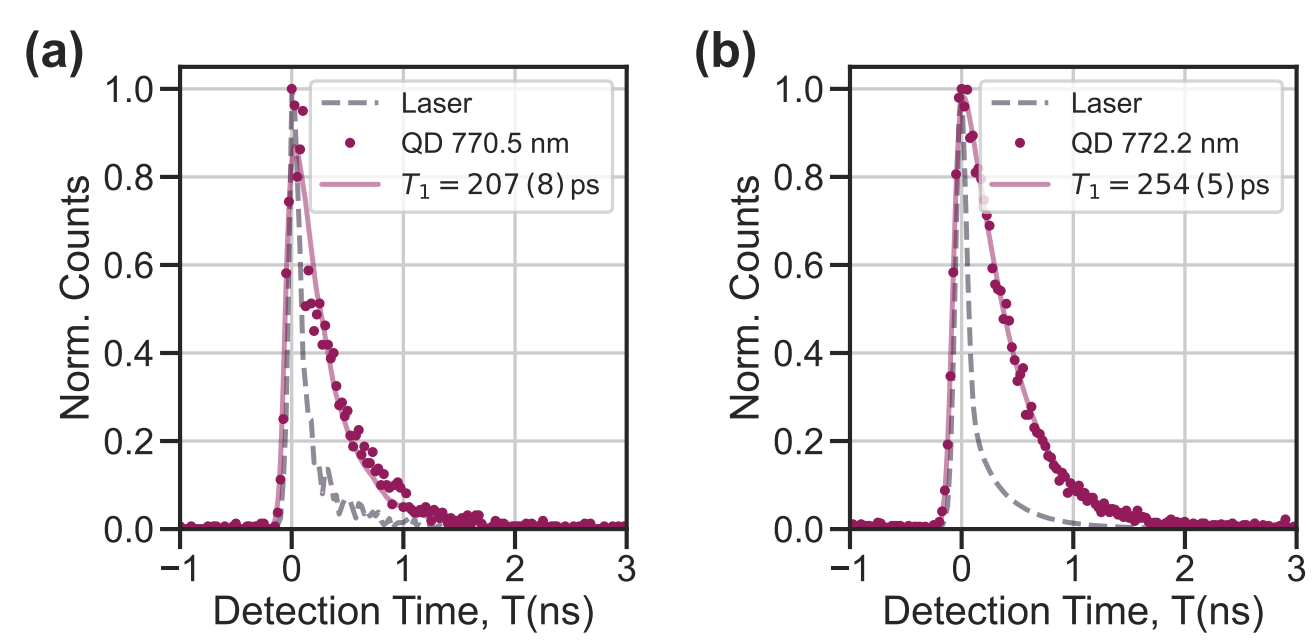

Supplementary Fig. 6. **Trion lifetime on Device 2. (a, b)** Trion lifetime, measured using a picosecond pulse laser, exciting resonantly and near resonant (phonon-assisted) in (a) and (b), respectively. The fits (solid-lines) are obtained by re-convoluting an exponential decay with the instrument-response function (dashed lines), giving a lifetime of $T_1 = 207\,(8)\,$ps and $T_1 = 254\,(5)\,$ps for the data in (a) and (b) respectively.

Supplementary Note 7. THREE-LEVEL MODEL FOR CPT

We start with the Hamiltonian of a three-level system,

$$\begin{aligned}\mathcal{H}_{3LS} = &\hbar\Delta\,|e\rangle\langle e| + \hbar\delta\,|\uparrow\rangle\langle\uparrow| \\ &+ \hbar\Omega_{\downarrow}/2\,(|\downarrow\rangle\langle e| + |e\rangle\langle\downarrow|) \\ &+ \hbar\Omega_{\uparrow}/2\,(|\uparrow\rangle\langle e| + |e\rangle\langle\uparrow|),\end{aligned}$$

where $|\downarrow\rangle$ and $|\uparrow\rangle$ are the two electron-spin ground states linked by a trion excited state $|e\rangle \equiv |\Downarrow\downarrow\uparrow\rangle$, $\delta = \omega - \omega_e^0$ is the detuning from two-photon resonance, and Δ is the single-photon detuning. The resonant Rabi frequencies for each transition from the excited state are labeled as $\Omega_{\downarrow}$ or $\Omega_{\uparrow}$. We include the collapse operators that define the ground-state relaxation Γ_1 and dephasing rate Γ_2, along with the spontaneous relaxation from the excited state along the spin-conserving $\gamma_{\mathrm{SC}} \approx \gamma_1$ and spin-flipping γ_{SP} channels as $\hat{C}_1 = \sqrt{\Gamma_1/2}\,(|\downarrow\rangle\langle\uparrow| + |\uparrow\rangle\langle\downarrow|)$, $\hat{C}_2 = \sqrt{\Gamma_2/2}\,(|\downarrow\rangle\langle\downarrow| - |\uparrow\rangle\langle\uparrow|)$, $\hat{C}_3 = \sqrt{\gamma_1}\,|\downarrow\rangle\langle e|$ and $\hat{C}_4 = \sqrt{\gamma_{\mathrm{SP}}}\,|\uparrow\rangle\langle e|$, respectively.

To fit the data, we fix $\Delta = 0$ and the ground and excited state relaxation rates to be $\Gamma_1^{-1} = 45000\,\text{ns}$, $\gamma_1^{-1} = 0.25\,\text{ns}$ and $\gamma_{\text{SP}}^{-1} = 100\,\text{ns}$. We then keep ω_e^0, $\Omega_\uparrow$, $\Omega_\downarrow$ and Γ_2 as free fitting parameters, and ω as our control axis. By fitting to the data in Fig. 2(b), we obtain $\omega_e^0/2\pi = 2.60(1)\,\text{GHz}$, $\Omega_\downarrow = 9.3(3)\,\text{ns}^{-1}$, $\Omega_\uparrow = 0.19(2)\,\text{ns}^{-1}$ and $\Gamma_2 = 0.53(8)\,\text{ns}^{-1}$. Given that the saturation Rabi frequency $\Omega_{\text{sat}} = \gamma_1/\sqrt{2} = 2.82\,\text{ns}^{-1}$, we can represent both Rabi frequencies in terms of Ω_{sat}, such that $\Omega_\downarrow = 3.29(9)\,\Omega_{\text{sat}}$ and $\Omega_\uparrow = 0.069(8)\,\Omega_{\text{sat}}$. The former is consistent with the pump power used in the experiment ($\approx 10\,\text{P}_{\text{sat}}$, hence $\Omega_\downarrow \approx \sqrt{10}\,\Omega_{\text{sat}}$) .

Supplementary Note 8. FOUR-LEVEL MODEL FOR RAMAN-DRIVEN RABI OSCILLATIONS

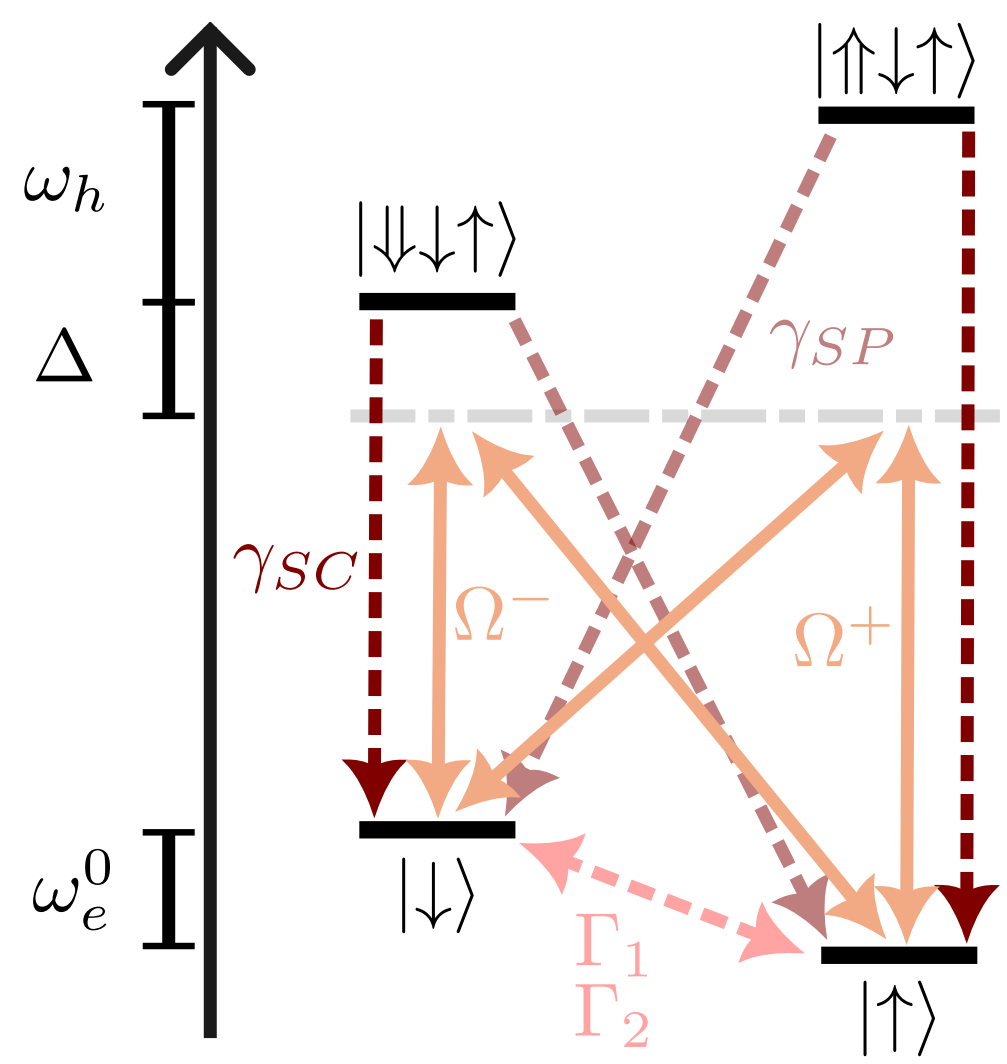

Supplementary Fig. 7. Four-level system. Solid arrows represent detuned Raman drive. Dashed arrows represent Lindblad operators, where we include the trion relaxation rates (γ_{SC} and γ_{SP}), along with the ground state electron relaxation (Γ_1) and dephasing (Γ_2) rates.

We consider a four-level system consisting of two spin states $|\uparrow\rangle$ and $|\downarrow\rangle$ in the ground state manifold, where a single electron occupies the QD. In the excited state manifold we account for two X^{1-} states $|\Uparrow\downarrow\uparrow\rangle$ and $|\Downarrow\downarrow\uparrow\rangle$. A sketch of the system is shown in Fig. Supplementary Fig. 7.

In Faraday geometry, the Hamiltonian of the system can be separated into the system Hamiltonian H_0 and the part accounting for the light-matter interaction H_{int} via

$$H = H_0 + H_{\text{int}}\,. \tag{S18}$$

We write the Hamiltonian in a rotating frame defined by the frequency of the first laser pulse, $E_{\text{trion}}/\hbar - \Delta$, resulting in the detuning Δ. In this frame, the second laser pulse appears with an additional detuning Δ_{RF}. The bare Hamiltonian is then given by

$$\begin{aligned} H_0 = 0\,|\downarrow\rangle\langle\downarrow| - \hbar\omega_e\,|\uparrow\rangle\langle\uparrow| \\ + \hbar\Delta\,|\Downarrow\downarrow\uparrow\rangle\langle\Downarrow\downarrow\uparrow| + \hbar\,(\Delta+\omega_h)\,|\Uparrow\downarrow\uparrow\rangle\langle\Uparrow\downarrow\uparrow|\,. \end{aligned} \tag{S19}$$

The light-matter interaction in standard rotating wave and dipole approximation reads

$$\begin{aligned} H_{\text{int}} = -\frac{\hbar}{2}\left(\Omega^-\,|\Downarrow\downarrow\uparrow\rangle\langle\downarrow| - \Omega^+\,|\Uparrow\downarrow\uparrow\rangle\langle\uparrow|\right) \\ - \frac{\hbar}{2}\frac{1}{\sqrt{\mathcal{C}}}\left(\Omega^-\,|\Downarrow\downarrow\uparrow\rangle\langle\uparrow| + \Omega^+\,|\Uparrow\downarrow\uparrow\rangle\langle\downarrow|\right) + h.c. \end{aligned} \tag{S20}$$

The cyclicity $\mathcal{C}$ is used to tune the ratio between strong spin-conserving and weak spin-flipping transitions. The left (Ω^-) and right (Ω^+) circularly polarized components of the envelope are given by

$$\Omega^- = \Omega_1 + \Omega_2 \exp^{-i\Delta_{RF}t} \tag{S21}$$

$$\Omega^+ = 0 \tag{S22}$$

to match the experimental configuration.

To describe relaxation processes from the trions to the ground states, we use Lindblad rates $\Gamma_i' \in \{\gamma_{SC}, \gamma_{SP}\}$ and their corresponding Lindblad operators L_i. We also employ Lindblad terms to account for a direct relaxation between both ground states at rate Γ_1. Furthermore, we take into account the dephasing of electron ground state coherences by exponentially damping them with a constant damping rate Γ_2. These processes are shown in Fig. Supplementary Fig. 7 by dashed arrows.

This leads to the equations of motions for a density matrix

$$\dot{\rho} = -\frac{i}{\hbar}\left[H,\rho\right] + \sum_i \Gamma_i'\left(L_i\rho L_i^\dagger - \frac{1}{2}\left\{L_i^\dagger L_i,\rho\right\}\right). \tag{S23}$$

We solve this equation numerically to calculate the dynamics of the system. To include the effect of the finite dephasing, evident from the decay time scale (T_2^* value) and the order (n=2) from the free-induction decay experiments, we perform a weighted average on $\rho(t,\omega)$, i.e.

$$\rho_{\text{ensemble}}(t) = \int w(\omega)\rho(t,\omega)\mathrm{d}\omega \tag{S24}$$

with the weight function modeled as a Gaussian noise with $\sigma = \sqrt{2}/T_2^*$,

$$w(\omega) = \frac{1}{\sigma\sqrt{2\pi}} \exp^{-\frac{1}{2}\left(\frac{\omega-\omega_e}{\sigma}\right)^2} \tag{S25}$$

is performed.

Using the parameters listed in Table Supplementary Table 1, we can match the output of the simulation with

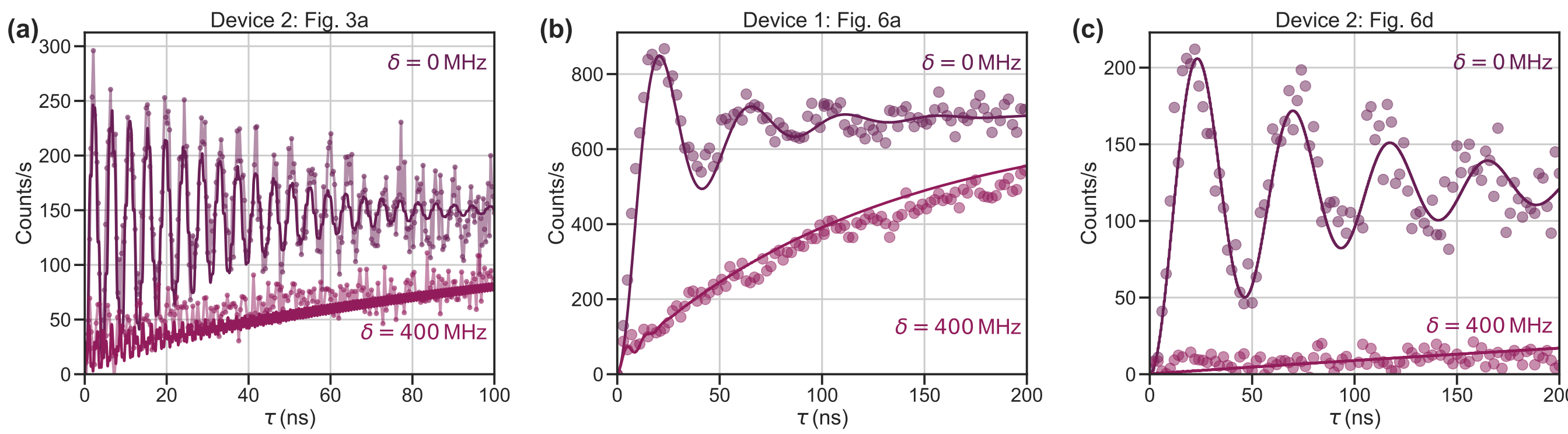

Supplementary Fig. 8. **Four-level Model Simulation. (a, b, c)** The output of the four-level model, on ($\delta = 0$) and off-resonance ($\delta \neq 0$), using the parameters in Supplementary Table 1 for the experimental data in Fig. 3(a), 6(a), and 6(d), respectively.

Supplementary Table 1. Parameters used to produce the fits in Fig. 3(a), 6(a) and 6(d).

**Dataset**	$\Omega/2\pi$ **(MHz)**	$\gamma_{SC}/2\pi$ **(MHz)**	$\gamma_{SF}/2\pi$ **(MHz)**	$\Gamma_1/2\pi$ **(MHz)**	$\Gamma_2/2\pi$ **(MHz)**	T_2^* **(ns)**	$\Delta/2\pi$ **(GHz)**
Fig. 3(a)	230	637	2.83	0.106	4.55	34	600
Fig. 6(a)	25	227	0.784	1.06	5.68	17	600
Fig. 6(d)	20	637	2.83	0.106	2.65	34	600

the experimental data in Fig. 3(a) and Fig. 6(a, d). As solving the four-level model is resource extensive, fitting of the experimental data in Fig. 3 and Fig. 6 is done with a two-level model [2, 10] instead. Both models result in similar values of the Rabi frequency Ω, ground state relaxation Γ_1 and pure dephasing Γ_2.

[1] R. Stockill, M. J. Stanley, L. Huthmacher, E. Clarke, M. Hugues, A. J. Miller, C. Matthiesen, C. Le Gall, and M. Atatüre, Phase-Tuned Entangled State Generation between Distant Spin Qubits, Physical Review Letters **119**, 010503 (2017).

[2] D. A. Gangloff, G. Éthier Majcher, C. Lang, E. V. Denning, J. H. Bodey, D. M. Jackson, E. Clarke, M. Hugues, C. Le Gall, and M. Atatüre, Quantum interface of an electron and a nuclear ensemble, Science **364**, 62–66 (2019).

[3] D. M. Jackson, D. A. Gangloff, J. H. Bodey, L. Zaporski, C. Bachorz, E. Clarke, M. Hugues, C. Le Gall, and M. Atatüre, Quantum sensing of a coherent single spin excitation in a nuclear ensemble, Nature Physics **17**, 585–590 (2021).

[4] R. H. J. Stockill, *Linking confined electron spins through coherent light-matter*, Ph.D. thesis, Cambridge (2017).

[5] M. Kroner, K. M. Weiss, B. Biedermann, S. Seidl, A. W. Holleitner, A. Badolato, P. M. Petroff, P. Öhberg, R. J. Warburton, and K. Karrai, Resonant two-color high-resolution spectroscopy of a negatively charged exciton in a self-assembled quantum dot, Physical Review B **78**, 075429 (2008).

[6] J. Wang, G. C. Guo, and L. He, Theory of strain tuning fine structure splitting in self-assembled inas/gaas quantum dots, Journal of Physics: Condensed Matter **26**, 475301 (2014).

[7] V. Mlinar, M. Tadić, B. Partoens, and F. M. Peeters, Nonsymmetrized hamiltonian for semiconducting nanostructures in a magnetic field, Phys. Rev. B **71**, 205305 (2005).

[8] J. Johansson, P. Nation, and F. Nori, QuTiP: An open-source Python framework for the dynamics of open quantum systems, Computer Physics Communications **183**, 1760 (2012).

[9] J. Johansson, P. Nation, and F. Nori, QuTiP 2: A Python framework for the dynamics of open quantum systems, Computer Physics Communications **184**, 1234 (2013).

[10] J. H. Bodey, R. Stockill, E. V. Denning, D. A. Gangloff, G. Éthier Majcher, D. M. Jackson, E. Clarke, M. Hugues, C. L. Gall, and M. Atatüre, Optical spin locking of a solid-state qubit, npj Quantum Information **5**, 95 (2019).

[11] M. J. Stanley, C. Matthiesen, J. Hansom, C. Le Gall, C. H. H. Schulte, E. Clarke, and M. Atatüre, Dynamics of a mesoscopic nuclear spin ensemble interacting with an optically driven electron spin, Phys. Rev. B **90**, 195305 (2014).

[12] M. Reindl, J. H. Weber, D. Huber, C. Schimpf, S. F. Covre da Silva, S. L. Portalupi, R. Trotta, P. Michler, and A. Rastelli, Highly indistinguishable single photons from incoherently excited quantum dots, Phys. Rev. B **100**, 155420 (2019).
