# Supplementary material for: Coherent control of quantum-dot spins with cyclic optical transitions
Source: Nat Commun. 2026 Jun 24;17:7917. doi: 10.1038/s41467-026-74590-z (PMC13444142; doi:10.1038/s41467-026-74590-z)
Supplement: Supplementary file 2 [file 41467_2026_74590_MOESM2_ESM.pdf]

nature portfolio

Peer Review File

Coherent Control of Quantum-Dot Spins with Cyclic Optical Transitions

Corresponding Author: Professor Dorian Gangloff

This file contains all reviewer reports in order by version, followed by all author rebuttals in order by version.

Attachments originally included by the reviewers as part of their assessment can be found at the end of this file.

Version 0:

Reviewer comments:

Reviewer #1

(Remarks to the Author)
The Authors demonstrate coherent control of a quantum dot (QD) electron spin in the Faraday configuration, for both GaAs (strain-free droplet-etched grown QD) and GaInAs (self-assembled QD). Their approach is based on stimulated Raman transitions with a two-photon detuning that accounts for the differential ac Stark shift induced by the strong difference in oscillator strength of the vertical vs diagonal transitions in this magnetic field configuration. The Authors then explore possible implementations of this capability. They study the quality factor of the induced Rabi oscillations and identify the best range of parameters, and point out the role of laser intensity fluctuations (or more accurately fluctuations of the Rabi frequency) on the quality factor. They implement Ramsey sequences to demonstrate full spin control over the Bloch sphere. They then demonstrate that magnon sidebands (quanta of the nuclear spin bath) are visible in ESR scans of the GaAs QD using a Rabi pulse of variable detuning. Those are also visible in spin echo spectra. Finally, the Authors perform a performance comparison of the two devices, which is in favour of the GaAs QD device.

This work solves a long-standing challenge for the use of QDs for quantum networks: the coherent manipulation of the electron spin in the Faraday configuration – the only configuration allowing for single-shot spin readout via cycling transitions. This ability opens a wide range of possible experiments in the context of quantum networks, with the appealing possibility to interface photons with long-lived nuclear spin memories in a device where efficient spin reading is available.

The experiments are delicate, and performed with a high rigour. The study is very exhaustive. It presents a convincing demonstration of coherent spin control for two kinds of QDs: GaAs QDs grown via droplet-etching epitaxy, and “standard” GaInAs self-assembled QDs. Moreover, the Authors provide evidence for a future implementation of an interface with nuclear spins, as testified by the demonstration of magnon sidebands in the Rabi spectrum.

I support publication in Nature Communications, provided that the Authors clarify the few following points.

- Regarding the estimation of the cyclicity (section II), the Authors provide error bars that seem to be obtained from the uncertainty on the spin pumping time only, as if the lifetime were certain. However, the Authors did not measure the emitter lifetime and estimate it from other QDs. Why did the Authors not measure the lifetime of the studied QDs? I suggest the Authors to include the uncertainty on the lifetime in their error calculation.

- The ac Stark shift decrease the energy of the two spin states. These energy shifts seems to be counted positively (as \Omega^2/4 \Delta) according to p.4, left column. The spin splitting and the two-photon resonance are also counted positively. For a sigma-minus laser, the splitting is decreased, so omega_e decreases (as shown later in the study). However, according to the expression \Delta \delta = \delta \downarrow - \delta \uparrow = \Omega_down^2 - \Omega_up^2 with \Omega_down >> Omega_up, we obtain that \Delta \delta is positive in this case, and therefore \omega_e = \omega_e^0 + \Delta\delta should be increased, where it is actually smaller. Maybe am I missing something but could the Authors verify and confirm the signs in the definitions.

- Could the Authors comment on their choice to keep the two Raman field intensities identical? Did the Authors consider compensating for the difference in oscillator strength by using a higher intensity for the vertical vs the diagonal component of

the stimulated Raman process?

- What limits the spin pumping fidelity on fig. 1 d and e? It seems that the reminiscent count rate is too high to be explained only by the spin T1.

- Why do the Authors alternate between Ramsey sequences with a pi phase difference of the second pulse instead of a single Ramsey sequence?

- The magnon sidebands are only visible in the positive detuning, due to a purely collinear interaction. The role of the small Zeeman splitting is mentioned, but could the Authors comment on the role of the magnetic field orientation, since in Voigt geometry both sidebands are observed for each nuclear spin specie in a similar QD (ref. 17).

- Could the Authors comment on the necessity of nuclear spin cooling for performing coherent control in Faraday geometry, beyond prolonging the spin T2*? As a comparison, optical spin rotations can be realised in Voigt geometry regardless of the spin T2*.

Reviewer #2

(Remarks to the Author)
Please find attached my review file.

Reviewer #3

(Remarks to the Author)
Manuscript title: Coherent Control of Quantum-Dot Spins with Cyclic Optical Transitions

This manuscript demonstrates full quantum control of an electron-spin qubit in a Faraday configuration, for the first time to my knowledge. Quantum dot spins in the Faraday configuration are known for the efficient spin-selective optical readout, but it is hard to achieve coherent control on the spins for the same reason. Using HH-LH mixing in the QD, the authors successfully bridge the two separate spin-selective optical transitions, showing full quantum control while maintaining a high cyclicity of 409. Overall, the results discussed in this manuscript are of high technical quality, with claims well supported by the data presented and methods commonly used in the same field. I believe that this work is of high impact not only to the QD community but also of interests to quantum network and quantum memory communities. After addressing a few minor points listed below, I would recommend publication of this manuscript in Nature Communications.

Specific Comments:
1. This study is carried out with a single electron state (x-). Is there a specific reason for choosing this state rather than the single hole state (x+)? I believe that, generally, hole spins have longer coherence times as they are subject to less nuclear-spin noise. Does the HH-LH mixing make it more complicated to study hole spins?
2. What are the knobs that can be used to tune the HH-LH mixing? In the conclusion, the authors mentioned strain as a knob for GaAs QDs. Can it be used for InGaAs QDs as well? How does the large built-in strain of InGaAs dots affect this?
3. In Fig. 1c, the authors provide a resonant spectroscopy map of a GaAs QD as a function of device bias and laser frequency. The contrast between the center and edges of the charge plateau appears weaker than in the InGaAs case (Fig. 1b). Could the authors comment on what might lead to this difference? Is it related to differences in the spin states of the two kinds of QDs? In addition, is the discontinuity in the QD trion line a measurement artifact? If so, I suggest the authors declare that in the figure caption.
4. Regarding the report of cyclicity, I find it surprising that the authors used previously reported values (by a different group in the case of GaAs QDs) rather than measured values for the QDs under study. Indeed, intrinsic QD lifetime can be mostly the same across different growth batches with similar heterostructures, but might be slightly different from different chambers. For example, the InAs QDs that I work with typically have lifetime around 1 ns, which is slightly longer than the reported value of 700 ps in the manuscript. Therefore the lifetime of the exact dots, or an averaged value of other dots on the same device, for an accurate claim of the cyclicity.
5. In Fig. 1a, is the label omega_laser intended to indicate the laser frequency used in spin initialization time measurements? If this is the case, I believe it is labeled at the wrong transition. Please double check this.

Version 1:

Reviewer comments:

Reviewer #1

(Remarks to the Author)
The Authors have satisfactorily addressed all my comments. I fully support publication of the revised manuscript.

Reviewer #2

(Remarks to the Author)
The authors have satisfactorily addressed my initial comments, as well as adding significant new theoretical details to the

paper. I am happy to recommend publication in Nature Communications.
A few minor final comments, however they do not impact the overall quality of the manuscript and I would recomment publication whether or not they are addressed:

- The authors now note a weaker red-detuned sideband in the magnon spectrum to explain how they achieve bidirectional feedback for Raman cooling (which in the data looks like it is essentially within the noise floor of their measurement). Can the authors say anything about the most likely mechanism generating this sideband? Residual strain, or some slight misalignment of the magnetic field axis relative to the sample? Currently the last paragraph of the "origins of nuclear cooling" section states both that they do not expect to see a non-collinear interaction, then a few sentences later effectively states that they do see a non-collinear interaction.

- In the supplementary materials (around Eq. S6 in the new version) the authors now state that "The spin flipping transitions ⇑h→↓ and ⇓h→↑ allowed by the χ term are circularly polarized and by the ϵ term πz polarized." I found the notation confusing here - are ⇑h and ⇓h in this sentence defined as in Eq. S6? If so, it seems to me like the change in angular momentum from the χ terms would be zero (e.g. for ⇑h as defined in Eq. S6, the χ* term is |3/2, -1/2>, so a transition from ⇑h→↓ would go from |3/2, -1/2> to |1/2, -1/2>, i.e. a change in the Z-projection of zero). Perhaps I am getting confused by the switch to the valence electron picture, but it would be good if the authors could clarify this.

Reviewer #3

(Remarks to the Author)
I thank the Authors for addressing my concerns in detail, and I think all modifications to the main text and supplementary appropriate. I especially like the revised Supplementary C, which adds great details that could potentially help this manuscript reach a broader audience. I recommend publication of this manuscript in its current form at Nature Communications.

Open Access This Peer Review File is licensed under a Creative Commons Attribution 4.0 International License, which permits use, sharing, adaptation, distribution and reproduction in any medium or format, as long as you give appropriate credit to the original author(s) and the source, provide a link to the Creative Commons license, and indicate if changes were made.
In cases where reviewers are anonymous, credit should be given to 'Anonymous Referee' and the source.
The images or other third party material in this Peer Review File are included in the article's Creative Commons license, unless indicated otherwise in a credit line to the material. If material is not included in the article's Creative Commons license and your intended use is not permitted by statutory regulation or exceeds the permitted use, you will need to obtain permission directly from the copyright holder.
To view a copy of this license, visit https://creativecommons.org/licenses/by/4.0/

Reply to the Reviewers

We thank the Reviewers for the time that they dedicated to reviewing our work. We are grateful for their careful evaluation of the manuscript and their thoughtful suggestions, and are happy to see that they assess our results positively.

Throughout this document *black-coloured italicised* font is used for the Reviewer comments and blue-coloured font is used for our replies. Our replies to each comment may include a list of associated changes to the manuscript. In our resubmission we include copies of the revised main text and supplemental material in which changes have been highlighted. The quoted reference numbers in the document below always refer to the revised documents. We refer to Appendices from the "diff" version of the revised manuscript for ease of viewing (these have been turned into a Methods section and a Supplementary Information file in the article files formatted for Nature Communications).

Reply to Reviewer #1

This work solves a long-standing challenge for the use of QDs for quantum networks: the coherent manipulation of the electron spin in the Faraday configuration – the only configuration allowing for single-shot spin readout via cycling transitions. This ability opens a wide range of possible experiments in the context of quantum networks, with the appealing possibility to interface photons with long-lived nuclear spin memories in a device where efficient spin reading is available.

The experiments are delicate and performed with a high rigour. The study is very exhaustive. It presents a convincing demonstration of coherent spin control for two kinds of QDs: GaAs QDs grown via droplet - etching epitaxy, and "standard" GaInAs self-assembled QDs. Moreover, the Authors provide evidence for a future implementation of an interface with nuclear spins, as testified by the demonstration of magnon sidebands in the Rabi spectrum.

I support publication in Nature Communications, provided that the Authors clarify the few following points.

1. *Regarding the estimation of the cyclicity (section II), the Authors provide error bars that seem to be obtained from the uncertainty on the spin pumping time only, as if the lifetime were certain. However, the Authors did not measure the emitter lifetime and estimate it from other QDs. Why did the Authors not measure the lifetime of the studied QDs? I suggest the Authors to include the uncertainty on the lifetime in their error calculation.*

   We thank the reviewer for pointing out this. Due to hardware limitations (detector timing jitter and lack of appropriate pulsed laser) at the time the work was conducted, we were unable to measure the trion lifetime for the particular

QDs we studied. For this reason, we estimated the lifetime from previous literature on the same or similar samples with similar emission wavelength.

We agree with the reviewer, however, that this is not the fairest representation of the uncertainty on our cyclicity. To provide a better estimate of this uncertainty, we have updated the manuscript with the following:

- For the GaAs QD, we have since been able to take measurements of the trion lifetime on 3 separate QDs of similar wavelength and sampled from three different pieces of the same wafer used for the manuscript QD. We obtained lifetimes of 207(8) ps, 254(5) ps, and 245(8) ps for QDs with a negatively charged trion emission of 770.5 nm, 772.2 nm, and 775.5 nm, respectively. This results in a wafer-wide estimate of the trion lifetime of 235(25) ps, which is the value we now use in the manuscript to estimate cyclicity and its uncertainty. These measurements are shown in a new Appendix H. We also note that the exciton lifetimes for GaAs QDs reported in the literature are remarkably consistent, around 250ps and vary little within a given wafer.
- For the InGaAs QD (our QD emits at 975 nm for the trion transition), the same device was used in Refs [45,46], where they reported the trion lifetimes of 5 QDs emitting near 970nm, leading to an estimate of our QD trion lifetime of 696 (65) ps. This is explained in Appendix H.

The values of cyclicity have been updated throughout the manuscript to reflect these mean lifetimes and their uncertainty.

2. *The ac Stark shift decrease the energy of the two spin states. These energy shifts seems to be counted positively (as \Omega^2/4 \Delta) according to p.4, left column. The spin splitting and the two-photon resonance are also counted positively. For a sigma-minus laser, the splitting is decreased, so omega_e decreases (as shown later in the study). However, according to the expression \Delta \delta = \delta \downarrow - \delta \uparrow = \Omega_down^2 - \Omega_up^2 with \Omega_down >> Omega_up, we obtain that \Delta \delta is positive in this case, and therefore \omega_e = \omega_e^0 + \Delta\delta should be increased, where it is actually smaller. Maybe am I missing something but could the Authors verify and confirm the signs in the definitions.*

   We thank the referee for spotting this. It is indeed a sign error in the definition. A decrease in energy of the state has indeed been defined as a positive AC Stark shift. We wish to keep the sign definition of the differential stark shift $\Delta\delta = \delta_{|\downarrow\rangle} - \delta_{|\uparrow\rangle}$ consistent with our detuning $\delta = \omega - \omega_e$, which implies that a positive shift should result from a decrease of ω_e. To achieve this the expression for the modified electron spin splitting should be $\omega_e = \omega_e^0 - \Delta\delta$. We have updated the

manuscript in a few places to reflect this change of sign (e.g. sign of eta) and Fig. 2a as a result.

We have also removed the absolute value symbols from Eq 2, as $\Delta\delta$ can of course be negative, corresponding to a value $\eta < 1$.

3. *Could the Authors comment on their choice to keep the two Raman field intensities identical? Did the Authors consider compensating for the difference in oscillator strength by using a higher intensity for the vertical vs the diagonal component of the stimulated Raman process?*

The reason for keeping our two Raman field intensities identical is two-fold.

First, we use a standard, off-the-shelf electro-optical intensity modulator, which generates phase-locked, symmetric sidebands when subject to sinusoidal microwave modulation. This makes the generation of amplitude and phase modulated pulse sequences straightforward with an AWG.

Second, while asymmetric Raman sidebands can be induced experimentally via phase-locking of two independent lasers or asymmetric sideband generation, compensating for a difference in oscillator strength is not as straightforward as it seems: if the dipole polarisation for the spin-conserving (stronger) and spin-flipping (weaker) transitions are mostly identical, as is the case for the GaAs QD measured in our experiment (c.f. Figure 2d and Figure 8c-e), sending asymmetric laser sidebands induces the same differential AC stark shift as with symmetric laser sidebands (for a fixed power). Strongly asymmetric sidebands with different polarisations could however work with the InGaAs QD, for which the distinct π_z component of the spin-flipping transition, c.f. Figure 10c, *which always addresses both spin states*, could allow for reducing the differential Stark shift. The circularly polarised leg of the Raman beams addressing the spin-conserving transition, which is spin selective, will however always create a differential Stark shift.

More elaborate schemes operating at very specific Raman detunings and trion splittings, or exploiting a third independent laser beam, could be exploited to truly cancel the differential Stark shift, but at the obvious cost of reduced flexibility and additional complexity.

To make this potential improvement towards compensating the differential AC Stark shift clear to the reader, we have updated the main text at the end of section III with the following sentence:

"If a π_z component is present in the spin-flipping transition (as we suppose is the case for the InGaAs QD but not the GaAs QD), then this shift could be mitigated, though not eliminated, using a Raman drive with two asymmetric sidebands."

4. *What limits the spin pumping fidelity on fig. 1 d and e? It seems that the reminiscent count rate is too high to be explained only by the spin T1.*

   We thank the reviewer for noticing this. While the spin pumping fidelity is indeed fundamentally limited by a competition of the spin T1 ($\Gamma_1^{-1} = 1/T_1$) with the transition rate of the spin flipping transition γ_{SP} (Phys. Rev. B 78, 075429 (2008)), time-resolved spin pumping measurement as a function of detuning, varied by changing the sample bias, shows that the steady-state count rate is dominated by off-resonant repumping by the readout laser at the powers we used for this data, which was well above saturation. We have included a new paragraph and figure in Appendix B, quantifying our spin pumping/ initialization fidelity, and have pointed the reader to it in the main text.

   In subsequent initialisations throughout the manuscript, we operate at lower power and, for the GaAs QD, at higher magnetic field (6.5T), which reduces the off-resonant scattering to become insignificant. Appendix B, Figure 8, shows the steady-state fluorescence to be limited by laser background in this situation, for both InGaAs and GaAs QDs. Using the spin pumping rate under these conditions, we can estimate the *upper bound* to the spin pumping fidelity, limited by spin T1, $F_{SP}^{upper} = (1 + 4\Gamma_1/\gamma_{SP})^{-1} = 0.97\ (0.98)$ for GaAs (InGaAs) QD based on the values measured in the experiment.

   We have added the following as clarification in the main text, at the end of section III: "The steady-state fluorescence at long delay time is dominated by off-resonant repumping of the spin state by the readout laser at these high powers. For this reason, in subsequent measurements presented in this article for both Devices 1 and 2, we perform spin initialisation at lower powers of $5P_{\rm sat}$ and $0.5P_{\rm sat}$ (and for Device 2 at a higher magnetic field) for which repumping is no longer significant and an initialisation fidelity upper bound of 98% and 97% can be reached, respectively (as detailed in Appendix B)".

5. *Why do the Authors alternate between Ramsey sequences with a pi phase difference of the second pulse instead of a single Ramsey sequence?*

   This is done to perform a balanced Ramsey measurement: in this way we read out the population of both electron spin states. We can then compute the Ramsey contrast directly, where a contrast of 1 (-1) denotes a final state of

down (up) state. This mitigates the effect of drifts in the count rate between measurement runs, allowing for robust determination of spin population. We have updated the manuscript to highlight this at the beginning of section V:

"This two-sequence configuration thus reads out both spin states at every delay time, providing a balanced readout."

6. *The magnon sidebands are only visible in the positive detuning, due to a purely collinear interaction. The role of the small Zeeman splitting is mentioned, but could the Authors comment on the role of the magnetic field orientation, since in Voigt geometry both sidebands are observed for each nuclear spin specie in a similar QD (ref. 17).*

   It is a non-collinear interaction which allows magnon sidebands of both positive and negative detunings (as seen in e.g. Ref 17). In strained QDs, a non-collinear interaction is present due to quadrupolar interactions, so long as the strain axis is not exactly co-aligned with the magnetic field (co-alignment is typically not possible due to strain inhomogeneity within a single QD) [Ref 14]. In strain-free QDs, the non-collinear interaction comes from g-factor anisotropy [Ref 16 and 17], and is present when an in-plane Voigt field is oriented at an angle with respect to GaAs crystal axes (but presumably also for a field with non-zero in-plane and out-of-plane components since in-plane and out-of-plane g-factors are different). For a pure out-of-plane Faraday field, however, as is the case here, there should not be any effect of g-factor anisotropy, and the non-collinear interaction should disappear. We observe that the magnon sidebands are indeed certainly dominant for positive detuning (Fig. 5a,b).

   We have made this clearer in section VI of the paper:
   "For a strain-free GaAs QD, a non-collinear interaction can arise from electronic g-factor anisotropy when the magnetic field is not aligned with the crystal axes of GaAs [Ref 16]. When subject to a growth-axis magnetic field, however, as is the case here, the electronic and nuclear quantization axes should be fully aligned and there should not be a significant non-collinear interaction."

7. *Could the Authors comment on the necessity of nuclear spin cooling for performing coherent control in Faraday geometry, beyond prolonging the spin T2*? As a comparison, optical spin rotations can be realised in Voigt geometry regardless of the spin T2*.*

   We agree that coherent optical spin rotations in the *Voigt* geometry can be realized without nuclear-spin cooling using either a detuned, ultrafast, pulsed laser (Nature **491**, 421-425 (2012)) or a stimulated Raman scheme that achieves very high Rabi frequency (c.f. Figure 1g in the Nature Physics **21**,

14475-1481 (2025), Ref[29]) to decouple from the uncooled electron T_2^* (of ~2 ns, c.f. Nature Communications 7, 12745 (2016) or FWHM of ESR of $1/T_2^*$ or ~$2\pi \times 250$ MHz). We note however that nuclear-spin cooling does not just make control much easier (by allowing phase-programmable Raman control at low Rabi frequency), it also **locks the value of the electron-spin resonance to a known and programmable value**, avoiding the deleterious effects of "dragging" (nuclear polarisation drifts and unwanted buildups) during complex pulse sequences. Nuclear-spin cooling is also required to narrow the ESR spectrum and flexibly probe nuclear spin-wave modes, and to enable continuous decoupling sequences (e.g. spin locking).

In the Faraday geometry, ultrafast pulsed laser control cannot work, since the optical pulses do not compete with the magnetic field as they do in Voigt geometry to instantaneously redefine the spin quantisation axis. Instead, it is necessary to spectrally resolve the ESR (and to compensate the differential Stark effect). In the parameter regime where we operate (which is typical) -- modest Rabi frequencies (i.e. much less than the electron-spin splitting), small electron-spin splitting (of a few GHz), and significant nuclear-spin interactions (noticeable nuclear dragging) – we have found it necessary to lock the nuclear polarisation, as well as to cool the nuclei and improve T2* to find the resonance conditions.

In section III, we have made a minor edit to clarify this:

"Finding the ESR frequency in a Raman experiment *typically* requires stabilizing the Overhauser shift imparted by nuclear spins on the electron-spin splitting [14]."

<u>Reply to Reviewer #2</u>

Overall, I found the manuscript to be a very impressive and complete piece of work. I do have a few questions and suggestions that I would appreciate if the authors could address, broken into a few different categories:

Questions about the setup

1. *Can the authors state more explicitly what the typical readout pulse duration used in their experiments is? The only information I could find regarding this is in Figs. 1(d),(e), which I believe is using significantly higher power than typically used for the actual readout pulses. This would be helpful as one drawback I see of using the Faraday scheme is that the readout pulses (e.g. in the quantum algorithmic cooling scheme) will take up more time.*

We thank the reviewer for pointing this out. We used a readout pulse duration of 2.5 us and 1.5 us for the measurements with InGaAs and GaAs QD, respectively. We have updated the manuscript to include the details on the duration of the readout and initialization pulses in all relevant figure captions.

2. *The fact that the authors can achieve such high spin Rabi frequencies for the GaAs device is interesting, especially given that according to Fig. 7(d) no solid immersion lens was used for the GaAs experiments. Could the authors add the input power required to reach the highest Rabi frequency, e.g. in Fig. 3? Perhaps I missed this information – I found some power information in Fig. 6, but it would be good to add in Fig. 3. Was an optical amplifier required to reach these Rabi frequencies?*

   We thank the reviewer for pointing this out. We used a power of ~300 uW on the cryostat window to achieve the Rabi frequency of 300 MHz for the GaAs QD. An optical amplifier is used to amplify the output from the electro-optical modulator to achieve such power in our setup. We have updated the section "Appendix A: Experimental Setup" to include the details of the optical amplifier, and the captions in Figure 3 to include the details of the input laser power at a Rabi rate of 250 MHz.

   Associated changes to the manuscript:

   Appendix A, We added the following sentence to describe our amplifier setup for the Raman laser.

   "To minimize the photo-refractive effect in our EOM that causes large fluctuations in the extinction ratio and drift in the EOM bias, we seed the EOM with a laser power of $\sim10\,$mW … We then amplify the signal with an optical amplifier (Thorlabs, BOA780) and spectrally filter the amplified signal with a grating filter (full-width-at-half-maximum of 35~GHz) to remove the broad amplified spontaneous emission from the amplifier."

3. *A related question – with no solid immersion lens in the GaAs device, would this mean less coupling to the component of the spin-flipping transition linearly polarised along the z-direction? Could this play some role in explaining why circular polarisation is optimal for spin control in the GaAs device, whereas for InGaAs the picture is more complex?*

   It is true that a solid-immersion lens (SIL) increases the numerical aperture of the Raman light incident on the QD and thus the component of the light linearly polarised along the (optical/field) z-axis. For the GaAs QD, we used an objective lens with NA=0.7. For the InGaAs QD, we used a 1mm cubic zirconia

SIL together an objective lens with NA = 0.5, which translates to an effective NA ~ 1.15 [Ref 68]. We have added NA information to Appendix A. Since NA ~ sin (divergence angle) ~ E_z, we expect the pi_z component in the GaAs case to be approximately 0.7/1.15 = 0.6 that of the InGaAs case; this is a relatively minor difference which we do not think can account for the absence of linearly polarised Raman coupling in the GaAs QD (Fig 2d).

Instead, we think the origin of the LH-HH mixing is fundamentally different for the GaAs vs InGaAs cases, whereby LHs of different spin dominate the mixing, respectively, leading to spin-flipping transitions with different polarisations. This is detailed in a revised and expanded Appendix C, and further discussed in multiple revised sentences in the main text (section III).

4. *The optical cyclicities are clearly very high, however if I understand correctly the numbers 289(3) and 409(6) are calculated using lifetime values measured for different quantum dots in the same sample. This makes the error bars on these estimates seem very low to me – was the error here estimated based only on the error in the measured optical spin pumping rate, with no error for the lifetime? If so, the error bars on these quantities are likely an underestimate.*

We thank the reviewer for pointing out this. Due to hardware limitations (detector timing jitter and lack of appropriate pulsed laser) at the time the work was conducted, we were unable to measure the trion lifetime for the particular QDs we studied. For this reason, we estimated the lifetime from previous literature on the same or similar samples with similar emission wavelength.

We agree with the reviewer, however, that this is not the fairest representation of the uncertainty on our cyclicity. To provide a better estimate of this uncertainty, we have updated the manuscript with the following:

- For the GaAs QD, we have since been able to take measurements of the trion lifetime on 3 separate QDs of similar wavelength and sampled from three different pieces of the same wafer used for the manuscript QD. We obtained lifetimes of 207(8) ps, 254(5) ps, and 245(8) ps for QDs with a negatively charged trion emission of 770.5 nm, 772.2 nm, and 775.5 nm, respectively. This results in a wafer-wide estimate of the trion lifetime of 235(25) ps, which is the value we now use in the manuscript to estimate cyclicity and its uncertainty. These measurements are shown in a new Appendix H. We also note that the exciton lifetimes for GaAs QDs reported in the literature are remarkably consistent, around 250ps and vary little within a given wafer.
- For the InGaAs QD (our QD emits at 975 nm for the trion transition), the same device was used in Refs [45,46], where they reported the trion

lifetimes of 5 QDs emitting near 970nm, leading to an estimate of our QD trion lifetime of 696 (65) ps. This is explained in Appendix H.

The values of cyclicity have been updated throughout the manuscript to reflect these mean lifetimes and their uncertainty.

Questions about the nuclear spin cooling:

5. *Could the authors add more complete information about the nuclear cooling sequences that were used? For example, in Fig. 4 Ω_{cool} and ω_{cool} are stated, but I couldn't find any information the actual duration of the cooling sequence. Same for Appendix F – some cooling parameters are given, but e.g. the duration of the entropy removal readout pulse is not stated. What is the total duration of the cooling cycle for the quantum algorithmic cooling? Does this take a lot longer than in the Voigt configuration, given that optical spin pumping is slower?*

   We have updated the manuscript to include the readout duration and the duration of the cooling sequence for the nuclear spin cooling sequence in Figures 2c, 3, 4, 5 and 10. The total duration of the quantum algorithmic cooling cycle is 55 us and 85 us for GaAs and InGaAs experiment, respectively. This is longer than the (quantum algorithmic) cooling time for experiments in the Voigt configuration as the optical spin pumping is slower (100 ns for experiment in Voigt configuration and ~2 us for our experiment in Faraday configuration). For instance, Phys. Rev. X. 12, 031014 (2022) [Ref 25] and Nature Physics 21, 1475-1481 (2025) [Ref 29]] reported 15 us of optimal cooling time and Nature Physics 21, 368-373 (2025) [Ref 17] reported 8 us of optimal cooling time.

6. *I find the authors' claim that the hyperfine interaction for the GaAs device in the Faraday geometry should be purely collinear very interesting. In Figure 5(a) the authors measure a magnon spectrum with only one sideband at positive detuning, which is stated in the text to be expected for a collinear hyperfine interaction. However, in Figure 4 the authors demonstrate that Raman cooling (as introduced by similar authors in Ref. [14]) works successfully in the GaAs device. If I understand correctly, the explanation for the cooling mechanism in Ref. [14] relies on a non-collinear hyperfine interaction to produce symmetric sidebands, such that nuclear spin flips in both directions away from the cooling lock-point can be corrected. Possibly I am missing something here, but can the authors explain why the Raman cooling works successfully for a purely collinear hyperfine interaction? Or does the fact that it works imply some non-collinear component?*

   This is a good question. We agree that Raman cooling can only work if bidirectional nuclear transitions are allowed from a single electron spin state

(i.e. it requires a non-collinear interaction). Our Figure 5 spectrum is dominated by the blue magnon sidebands due to a dominant collinear interaction. However, a red sideband remains present, as is clearest in Fig. 5b at d=-48MHz (the red Arsenic sideband). In this situation, the Raman cooling feedback curve (as per Ref [14]) is asymmetric but retains a stable lock-point -- as was the case, e.g. in Nature Physics 17, 1247 (2021) [Ref 58] where the large sideband asymmetry was due to nuclear polarisation, but Raman cooling remained stable. This is also the reason why we modify the quantum algorithmic cooling sequence to introduce a conditional nuclear spin flip (on the electron spin state) as described in Appendix F.

We have added a sentence in the main text at the end of section VI clarifying this point:

"We note the presence of a weaker red-detuned ($\delta < 0$) sideband near the As frequency (Fig.5a,b), which allows some degree of bidirectional feedback under the Raman cooling configuration (Fig.~4) despite the asymmetry [Ref 56]".

7. *A related question – was it possible to perform cooling at a lower magnetic field than 6.5T for the GaAs device? Based on the collinear hyperfine interaction explanation, I would expect the flip-flop hyperfine terms to become larger at lower magnetic fields (in accordance with the $1/\omega_e$ dependence stated by the authors). Presumably this would be apparent in the cooling parameters (e.g. perhaps a shorter τ_{hh}) or the magnon spectrum (e.g. the magnon sideband to appear for shorter Rabi drive times). Did the authors attempt any experiments like this at lower magnetic field?*

   We do not have a systematic investigation as a function of field strength. However, in practice, we found it difficult to operate at significantly lower field. It is likely that the combination of small g-factor (ω_e^0 only ~1GHz at 3.5T) and the large differential Stark shifts during Raman (up to 1GHz) make it difficult to stabilise the electron spin at lower fields, perhaps also in combination with the larger collinear hyperfine interaction which is indeed expected to scale as $1/\omega_e$.

   That said, QDs with larger g-factors, as is easily possible with GaAs QDs of slightly bluer, or significantly redder, wavelength (PRX Quantum 6, 040309 (2025), [Ref 32]), would allow experiments at lower fields.

More general questions:

8. *In Ref. [31], a waveguide structure is used to engineer the optical selection rules in the Voigt geometry and generate an optical cyclicity of 36. In Ref. [31], the optical cyclicity is not the major infidelity contribution for creating photonic*

cluster states, which is instead limited by unwanted off-resonant excitation of an optical transition that is not the target transition for single photon emission. Would the authors expect off-resonant excitation errors to be less in the Faraday configuration? I can imagine that a larger g-factor along the growth direction would result in more widely separated transitions.

As far as we understand Ref [31], the unwanted off-resonant excitation is co-polarised with the desired transition (x-dipole); these are also quite close in energy as they are the diagonal transitions in a system with $g_e < 0, g_h > 0$.

In Faraday, there are two dominant cross-polarised circular dipoles, which naturally protects against unwanted off-resonant excitation. A much smaller off-resonant excitation is possible via the weak spin-flipping transition, which is co-polarised for the GaAs QD, but is suppressed in a fast (i.e. coherent) resonant pi-pulse excitation by the cyclicity C~470 since the off-resonant pathway scattering will be, for Omega*t=pi, approximately sin(Omega*t/sqrt(C))^2 = sin(pi/22)^2 = 2%. This error is thus reduced, linearly, by a large cyclicity C. This is an upper bound on the error, as the detuning added by the electron-spin splitting can further reduce this error. If the weak spin-flipping transition is dominated by a π_z transition (as we think is the case for the InGaAs QD), then the off-resonant pathway is further suppressed by selection rules.

We've made a minor update to the introduction to reflect this discussion:
"Under a growth-axis field (Faraday configuration), the presence of spin-specific circularly-polarized cyclic transitions allows for efficient spin-state read-out [21, 22] in the single-shot regime [23, 24]"

9. *Are there any disadvantages that the authors see to using the Faraday configuration for optical cluster state generation?*

While we demonstrated coherent control of an electron spin together with a large optical cyclicity C, both critical for optical cluster state generation, the main drawback that we see, at the moment, is the reduced spin control fidelity. We achieved, with this GaAs QD, 97.4% π-pulse contrast (Q-factor of ~20), as compared with the highest π-pulse contrast reported in Voigt configuration of 99.3% [Ref 18]. Firstly, the larger optical power (by $\sqrt{C}$) required increases the power-dependent laser induced spin relaxation [Ref 27] for a fixed spin Rabi frequency. However, we have already witnessed improvements in this laser-induced spin relaxation the GaAs QD (Fig. 6b vs e), with further improvements possible. Secondly, the differential Stark shift (c.f. the inset in Figure 2a), which also scales as $\sqrt{C}$, can convert Raman laser intensity noise into decoherence. Neither are fundamental issues and we foresee near-term improvements on both accounts.

Reply to Reviewer #3

This manuscript demonstrates full quantum control of an electron-spin qubit in a Faraday configuration, for the first time to my knowledge. Quantum dot spins in the Faraday configuration are known for the efficient spin-selective optical readout, but it is hard to achieve coherent control on the spins for the same reason. Using HH-LH mixing in the QD, the authors successfully bridge the two separate spin-selective optical transitions, showing full quantum control while maintaining a high cyclicity of 409. Overall, the results discussed in this manuscript are of high technical quality, with claims well supported by the data presented and methods commonly used in the same field. I believe that this work is of high impact not only to the QD community but also of interests to quantum network and quantum memory communities. After addressing a few minor points listed below, I would recommend publication of this manuscript in Nature Communications.

Specific Comments:

1. *This study is carried out with a single electron state (x-). Is there a specific reason for choosing this state rather than the single hole state (x+)? I believe that, generally, hole spins have longer coherence times as they are subject to less nuclear-spin noise. Does the HH-LH mixing make it more complicated to study hole spins?*

   It is true that hole spins have longer coherence times compared to the electron thanks to the weaker hyperfine interaction (typically by a factor of about 10). But this also means that the coupling with the nuclear magnons is ~10x slower – the two go hand in hand – bringing the timescales closer to another important limit, T1, which does not change with hyperfine. Our reason for selecting the electron spin is thus simple, it is “10x better” if one wishes to establish a quantum repeater node, where the strong interactions between the electron and the nuclear ensembles allows for quantum information transfer between the electron and a nuclear magnon, effectively enabling a multi-qubit register within the same QD (Nature Physics 21, 368-373 (2025), [Ref 17]).

   Secondly, while nuclear spin cooling and nuclear magnons have been observed for hole-spins (Nature Physics 21, 1475-1481 (2025), [Ref 29]), the magnitude of the non-collinear term in the hole-spin hyperfine interactions is more complicated to predict and control, as it varies with HH-LH mixing, resulting in variation in interaction strengths for various QD. This makes studying hole-spins more complicated for spin-experiments. Further information regarding the nature of the HH-LH mixing for GaAs and InGaAs QDs can be found in the Appendix C and in Ref 32.

2. *What are the knobs that can be used to tune the HH-LH mixing? In the conclusion, the authors mentioned strain as a knob for GaAs QDs. Can it be used for InGaAs QDs as well? How does the large built-in strain of InGaAs dots affect this?*

   HH-LH mixing can be modified by strain (from lattice mismatch or via external actuation) and asymmetry in the geometry of the QD [Ref 32]. One can engineer up to ~2% homogeneous strain using external strain actuators [Ref 63] to vary the HH-LH mixing, which works well for strain-free GaAs (Nature Physics **10**, 46–51 (2014)) QDs. However, the achievable strains are significantly lower than the built-in strain of up to 10% in self-assembled InGaAs dots [Phys. Rev. B 85, 115313 (2012)]. We thus expect external strain tuning to be much more difficult for InGaAs dots.

   We have have added clarification that only few-percent strain is realistic in the relevant sentence in the Conclusions, and added a relevant reference (Ref 63).

3. *In Fig. 1c, the authors provide a resonant spectroscopy map of a GaAs QD as a function of device bias and laser frequency. The contrast between the center and edges of the charge plateau appears weaker than in the InGaAs case (Fig. 1b). Could the authors comment on what might lead to this difference? Is it related to differences in the spin states of the two kinds of QDs? In addition, is the discontinuity in the QD trion line a measurement artifact? If so, I suggest the authors declare that in the figure caption.*

   We thank the reviewer for noticing this. Our 2D maps reflects steady-state fluorescence for each value of bias and laser frequency (i.e. our bias ramp is slow compared to dynamics). The contrast between the center and edges of the plateau is thus determined by a competition of spin pumping and repumping (via off-resonant excitation) or relaxation (spin T1) in setting up a steady state. The spin T1 for both QD types is >40us in the center of the plateau, so this is not the reason for reduced contrast. Rather, because of the small spin splitting of the GaAs QD (~1GHz at 3.5T), repumping from off-resonant scattering will occur at moderate powers (and above) and a bias scan will always reveal some bias at which one gets significant steady-state fluorescence (This was already observed for a similar map on a GaAs QD with low splitting in Ref [18]). We realized thanks to this query that the 2D maps were actually taken well above saturation – 2Psat for the InGaAs QD and 8Psat for the GaAs QD.

   To address this in more detail for the reader we have added a Figure (8) to Appendix B, highlighting the striking difference in steady-state fluorescence at powers above saturation when the bias is set to off-resonant for the two types

of QDs. We have also added a sentence in the main text explaining this, and updated the caption to Figure 1.

The discontinuity in the QD trion line in Figure 1c is most likely due to the same physics: in the middle of the plateau the highest steady-state fluorescence appears at a bias which maximises off-resonant pumping and repumping, whereas at the edges where spin relaxation is much faster than optical repumping it appears at a bias corresponding to resonant scattering. Similar though less marked features can be seen on all branches on the approach to the co-tunnelling zones. We have added a sentence in the caption of Fig.1 to explain this. It is possible that nuclear spin polarisation could also play a role in building up hysteretic effects at these biases, but this would need further experimental investigation which isn't directly relevant to this work.

4. *Regarding the report of cyclicity, I find it surprising that the authors used previously reported values (by a different group in the case of GaAs QDs) rather than measured values for the QDs under study. Indeed, intrinsic QD lifetime can be mostly the same across different growth batches with similar heterostructures but might be slightly different from different chambers. For example, the InAs QDs that I work with typically have lifetime around 1 ns, which is slightly longer than the reported value of 700 ps in the manuscript. Therefore, the lifetime of the exact dots, or an averaged value of other dots on the same device, for an accurate claim of the cyclicity.*

   We thank the reviewer for pointing out this. Due to hardware limitations (detector timing jitter and lack of appropriate pulsed laser) at the time the work was conducted, we were unable to measure the trion lifetime for the particular QDs we studied. For this reason, we estimated the lifetime from previous literature on the same or similar samples with similar emission wavelength.

   We agree with the reviewer, however, that this is not the fairest representation of the uncertainty on our cyclicity. To provide a better estimate of this uncertainty, we have updated the manuscript with the following:

   - For the GaAs QD, we have since been able to take measurements of the trion lifetime on 3 separate QDs of similar wavelength and sampled from three different pieces of the same wafer used for the manuscript QD. We obtained lifetimes of 207(8) ps, 254(5) ps, and 245(8) ps for QDs with a negatively charged trion emission of 770.5 nm, 772.2 nm, and 775.5 nm, respectively. This results in a wafer-wide estimate of the trion lifetime of 235(25) ps, which is the value we now use in the manuscript to estimate cyclicity and its uncertainty. These measurements are shown in a new Appendix H. We also note that the exciton lifetimes for GaAs QDs reported

in the literature are remarkably consistent, around 250ps and vary little within a given wafer.

- For the InGaAs QD (our QD emits at 975 nm for the trion transition), the same device was used in Refs [45,46], where they reported the trion lifetimes of 5 QDs emitting near 970nm, leading to an estimate of our QD trion lifetime of 696 (65) ps. This is explained in Appendix H.

The values of cyclicity have been updated throughout the manuscript to reflect these mean lifetimes and their uncertainty.

5. *In Fig. 1a, is the label omega_laser intended to indicate the laser frequency used in spin initialization time measurements? If this is the case, I believe it is labelled at the wrong transition. Please double check this.*

   We thank the reviewer for noticing this. Yes, indeed, it was used to refer to the laser frequency used for the spin initialization measurements. This was indeed mislabelled and we have corrected this in the revised manuscript.

   Associated changes to the manuscript:

   - Main text, page 3, Figure 1(a). The symbol ω_{laser} is labelled on the lowest frequency transition, rather than the highest frequency transition in the previous version of the manuscript.
   - Main text, page 3, Figure 1, captions. We have added the text ", indicated by the red arrow in (a), ".

Other modifications

(1) We have significantly expanded Appendix C with a more detailed derivation of HH-LH mixing and its effect on Raman transitions in Faraday. We feel this will add significant value for the interested reader. This expanded discussion includes a prediction of the dependence of the HH-LH mixing on magnetic field strength, which provides an explanation for the asymmetric response we observed in Fig 2 to right- vs left-circularly polarised light. This is now highlighted in a new main text paragraph in section III.

These additions were made possible by our theory collaborator Dr Michał Gawełczyk, and have thus added them as an author.

(2) All three referees (rightfully) asked about our estimates of cyclicity from literature values of lifetime. As per our response to each referee, we have made new measurements of the trion lifetime on GaAs QDs from the same wafer, and have looked for measurements of the trion lifetime on InGaAs QDs of a similar wavelength on the same device, thus providing a much improved expectation value and uncertainty on this value.

The additional measurements were in part made possible by our team member Dr Yusuf Karli, and thus have added them as an author.

(3) We have updated the fits to the Rabi flop data in 3a, 6a and 6d, with the simulation curve. The parameters are listed in Appendix J: Fit parameters for the Rabi Flop data.

<u>**Reply to the Referees**</u>

We thank the referees for the time that they dedicated to our work. We are grateful for the careful evaluation of the manuscript and are happy to see that all 3 referees support the publication of our revised manuscript.

Throughout this document, italic, black-coloured font is used for the Referee's comments and blue-coloured font is used for our replies. Our replies to each comment include a list of associated changes to the manuscript. In our resubmission we include copies of the revised main text and supplemental material in which changes have been highlighted using red-coloured font.

<u>Reviewer #2</u>

1. *The authors now note a weaker red-detuned sideband in the magnon spectrum to explain how they achieve bidirectional feedback for Raman cooling (which in the data looks like it is essentially within the noise floor of their measurement). Can the authors say anything about the most likely mechanism generating this sideband? Residual strain, or some slight misalignment of the magnetic field axis relative to the sample? Currently the last paragraph of the "origins of nuclear cooling" section states both that they do not expect to see a non-collinear interaction, then a few sentences later effectively states that they do see a non-collinear interaction.*
   We thank the reviewer for the comment. We are unsure about origin at this stage, but agree with the reviewer that it could be residual strain or slight misalignment of the magnetic field. We have added a clarification to the relevant sentence:

   "We note the presence of a weaker red-detuned ($\delta < 0$) sideband near the As frequency (Fig.5a,b) – suggesting a small non-collinear interaction is present, possibly due to residual strain or to a slight misalignment of the magnetic field with respect to the device -- which allows some degree of bidirectional feedback under the Raman cooling configuration (Fig.4), despite the asymmetry~\cite{Gangloff2021}."

2. In the supplementary materials (around Eq. S6 in the new version) the authors now state that "The spin flipping transitions ⇑h→↓ and ⇓h→↑ allowed by the χ term are circularly polarized and by the ϵ term πz polarized." I found the notation confusing here - are ⇑h and ⇓h in this sentence defined as in Eq. S6? If so, it seems to me like the change in angular momentum from the χ terms would be zero (e.g. for ⇑h as defined in Eq. S6, the χ* term is |3/2, -1/2>, so a transition from ⇑h→↓ would go from |3/2, -1/2> to |1/2, -1/2>, i.e. a change in the Z-projection of zero). Perhaps I am getting confused by the switch to the valence electron picture, but it would be good if the authors could clarify this.

We thank the reviewer for pointing this out. The |⇑h> and |⇓h> were mistakenly swapped in Eq. (C6). To make this part more comprehensible, we decided to keep only valence kets in Eq. (C6) and add the following text below:
"To relate this to the hole picture, we use the convention that the hole spinor associated with a missing valence electron is the time-reversed spinor of that electron. Thus, e.g., a missing |⇑> electron corresponds to |⇓>_h = T|⇑>, which has formally the same spinor expansion as |⇓>"
We have also slightly rephrased the introduction of the valence-electron to hole correspondence at the beginning of Appendix C, which now reads:
"Note that (solely) in this Appendix C 1, we work in the valence electron picture. When such a valence-electron state is removed, the corresponding hole spinor used elsewhere in the manuscript is defined as its time-reversed spinor: |i⟩_h = T |i⟩, where T |J, Jz⟩ = (−1)^(J−Jz) |J, −Jz⟩."

The manuscript by Koong et al. demonstrates all-optical coherent control of single electron spins in both GaAs and InGaAs quantum dots in a magnetic field configuration that gives rise to highly cyclic optical transitions. Semiconductor quantum dots have exceptional photonic properties, and their spin coherence has been steadily improving, making them promising candidates for spin-photon interfaces. However, a major drawback for deploying quantum dots in quantum optical networks has been the challenge of integrating coherent spin control with single-shot readout. Optical single-shot readout requires a system with high cyclicity, such that the spin state is preserved when optically driving the readout transition. This work by Koong et al. shows for the first time a quantum dot system that combines coherent spin control with cyclic optical transitions. In my opinion, this is a very impressive piece of work that will be of interest to a broad range of readers and certainly deserves to be published in Nature Communications.

The key result that the authors demonstrate is all-optical spin control for a magnetic field applied along the quantum dot growth direction (Faraday geometry). To date all other demonstrations of optical spin control in quantum dots have used a magnetic field applied perpendicular to the growth direction (Voigt geometry), which does not permit a cyclic transition appropriate for single-shot readout. A major challenge for optical spin control in the Faraday configuration is that the imbalanced optical transitions lead to an unavoidable differential AC Stark shift of the optical transitions, resulting in a spin resonance frequency that depends on the rotation laser optical power. The authors show that with careful calibration this can be mitigated, although fluctuations in rotation laser power introduce a source of noise in the spin resonance frequency that is significantly worse than for the Voigt geometry. Nevertheless, the authors show the spin control works relatively well, with a maximum pi-pulse contrast of 97.4% for the GaAs device. The authors also successfully demonstrate nuclear spin cooling techniques in the Faraday configuration, which is a crucial step for extending the spin coherence to a potentially useful level. Single nuclear magnon spectroscopy is performed, suggesting that protocols previously demonstrated by the same group could also be used in the Faraday configuration.

Overall, I found the manuscript to be a very impressive and complete piece of work. I do have a few questions and suggestions that I would appreciate if the authors could address, broken into a few different categories:

Questions about the setup

1. Can the authors state more explicitly what the typical readout pulse duration used in their experiments is? The only information I could find regarding this is in Figs. 1(d),(e), which I believe is using significantly higher power than typically used for the actual readout pulses. This would be helpful as one drawback I see of using the Faraday scheme is that the readout pulses (e.g. in the quantum algorithmic cooling scheme) will take up more time.
2. The fact that the authors can achieve such high spin Rabi frequencies for the GaAs device is interesting, especially given that according to Fig. 7(d) no solid immersion lens was used for the GaAs experiments. Could the authors add the input power required to reach the highest Rabi frequency, e.g. in Fig. 3? Perhaps I missed this information – I found some power information in Fig. 6, but it would be good to add in Fig. 3. Was an optical amplifier required to reach these Rabi frequencies?
3. A related question – with no solid immersion lens in the GaAs device, would this mean less coupling to the component of the spin-flipping transition linearly polarised along the z-direction? Could this play some role in explaining why circular polarisation is optimal for spin control in the GaAs device, whereas for InGaAs the picture is more complex?

4. The optical cyclicities are clearly very high, however if I understand correctly the numbers 289(3) and 409(6) are calculated using lifetime values measured for different quantum dots in the same sample. This makes the error bars on these estimates seem very low to me – was the error here estimated based only on the error in the measured optical spin pumping rate, with no error for the lifetime? If so, the error bars on these quantities are likely an underestimate.

Questions about the nuclear spin cooling:

5. Could the authors add more complete information about the nuclear cooling sequences that were used? For example, in Fig. 4 Ω_{cool} and ω_{cool} are stated, but I couldn't find any information the actual duration of the cooling sequence. Same for Appendix F – some cooling parameters are given, but e.g. the duration of the entropy removal readout pulse is not stated. What is the total duration of the cooling cycle for the quantum algorithmic cooling? Does this take a lot longer than in the Voigt configuration, given that optical spin pumping is slower?
6. I find the authors' claim that the hyperfine interaction for the GaAs device in the Faraday geometry should be purely collinear very interesting. In Figure 5(a) the authors measure a magnon spectrum with only one sideband at positive detuning, which is stated in the text to be expected for a collinear hyperfine interaction. However, in Figure 4 the authors demonstrate that Raman cooling (as introduced by similar authors in Ref. [14]) works successfully in the GaAs device. If I understand correctly, the explanation for the cooling mechanism in Ref. [14] relies on a non-collinear hyperfine interaction to produce symmetric sidebands, such that nuclear spin flips in both directions away from the cooling lock-point can be corrected. Possibly I am missing something here, but can the authors explain why the Raman cooling works successfully for a purely collinear hyperfine interaction? Or does the fact that it works imply some non-collinear component?
7. A related question – was it possible to perform cooling at a lower magnetic field than 6.5T for the GaAs device? Based on the collinear hyperfine interaction explanation, I would expect the flip-flop hyperfine terms to become larger at lower magnetic fields (in accordance with the $1/\omega_e$ dependence stated by the authors). Presumably this would be apparent in the cooling parameters (e.g. perhaps a shorter τ_{hh}) or the magnon spectrum (e.g. the magnon sideband to appear for shorter Rabi drive times). Did the authors attempt any experiments like this at lower magnetic field?

More general questions:

8. In Ref. [31], a waveguide structure is used to engineer the optical selection rules in the Voigt geometry and generate an optical cyclicity of 36. In Ref. [31], the optical cyclicity is not the major infidelity contribution for creating photonic cluster states, which is instead limited by unwanted off-resonant excitation of an optical transition that is not the target transition for single photon emission. Would the authors expect off-resonant excitation errors to be less in the Faraday configuration? I can imagine that a larger g-factor along the growth direction would result in more widely separated transitions.
9. Are there any disadvantages that the authors see to using the Faraday configuration for optical cluster state generation?
